# Probing Multiscale Disorder in Pyrochlore and Related Complex Oxides in the Transmission Electron Microscope: A Review

**DOI:** 10.3389/fchem.2021.743025

**Published:** 2021-11-29

**Authors:** Jenna L. Wardini, Hasti Vahidi, Huiming Guo, William J. Bowman

**Affiliations:** ^1^ Materials Science and Engineering, University of California, Irvine, Irvine, CA, United States; ^2^ Irvine Materials Research Institute, Irvine, CA, United States

**Keywords:** disordered crystals, scanning transmission elctron microscopy, 4D-STEM, electron diffraction, order – disorder transformations: ODTs, amorphous materials, complex oxides

## Abstract

Transmission electron microscopy (TEM), and its counterpart, scanning TEM (STEM), are powerful materials characterization tools capable of probing crystal structure, composition, charge distribution, electronic structure, and bonding down to the atomic scale. Recent (S)TEM instrumentation developments such as electron beam aberration-correction as well as faster and more efficient signal detection systems have given rise to new and more powerful experimental methods, some of which (e.g., 4D-STEM, spectrum-imaging, in situ/operando (S)TEM)) facilitate the capture of high-dimensional datasets that contain spatially-resolved structural, spectroscopic, time- and/or stimulus-dependent information across the sub-angstrom to several micrometer length scale. Thus, through the variety of analysis methods available in the modern (S)TEM and its continual development towards high-dimensional data capture, it is well-suited to the challenge of characterizing isometric mixed-metal oxides such as pyrochlores, fluorites, and other complex oxides that reside on a continuum of chemical and spatial ordering. In this review, we present a suite of imaging and diffraction (S)TEM techniques that are uniquely suited to probe the many types, length-scales, and degrees of disorder in complex oxides, with a focus on disorder common to pyrochlores, fluorites and the expansive library of intermediate structures they may adopt. The application of these techniques to various complex oxides will be reviewed to demonstrate their capabilities and limitations in resolving the continuum of structural and chemical ordering in these systems.

## Introduction

Crystalline materials are generally thought of as highly-ordered systems, however, this classification is not fully descriptive without a reference to the length-scale of ordering. Although crystals display long-range structural and chemical periodicity, they also commonly host various types of local imperfections which we generally categorize as ‘disorder’. These local deviations from long-range order can include non-repeating variations in composition, atom displacements, bonding arrangements, molecular orientations, conformations, charge states, orbital occupancies, and/or magnetic structure (Simonov and Goodwin, 2020). Further, rather than being random in nature, these disruptions of the long-range periodicity are more often correlated, or locally-ordered on short (sub-unit-cell) to medium (few-unit-cell) length scales. As the connection between the structure, chemistry, and properties of materials across length scales becomes clearer, it is recognized that many interesting phenomena can only be understood by embracing the role of such disorder (Chaney et al., 2021). Multi-cation, or complex oxides are an ideal class of materials to study such phenomena, since their structure-property relationships are not only related to the composition and structure, but are correlated to the extent of disorder within the cation and anion sublattices (Kreller and Uberuaga, 2021; Mullens et al., 2021).

In this review, we present a suite of high spatial resolution electron microscopy characterization techniques that are uniquely suited to probe the many types, length-scales, and degrees of disorder in complex oxides, with a focus on disorder common to isostructures of the minerals pyrochlore (A_2_B_2_O_7_, Fd $\bar{3}$ m) and fluorite (BO_2_, Fm $\bar{3}$ m). These isometric mixed-metal oxides represent two ends of an expansive library of intermediate structures that encompass varying degrees of disorder on their cationic and anionic sublattices (Mullens et al., 2021). Disorder is adopted during disordering transitions, often in lieu of long-range symmetry-lowering transformations away from their cubic parent phase. This makes them ideal structures to host complex types of disorder (and/or local order) that can profoundly affect their functional properties (Subramanian et al., 1983; Maram et al., 2018), which are quite diverse. Here, we focus on their high radiation tolerance (Sickafus et al., 2000) and high ionic conductivity (Jitta et al., 2015; Kim et al., 2020), two properties that make them ideal candidate materials for nuclear waste management applications (Ewing et al., 2004; Orlova and Ojovan, 2019) and as solid-oxide fuel cell components (Anantharaman and Dasari, 2021).

### Pyrochlores, Fluorites and Intermediate Structures

**FIGURE 1:**
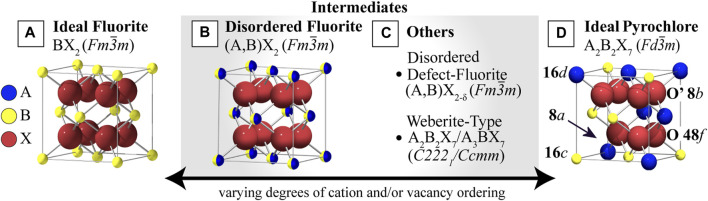
Fluorite, Pyrochlore, and intermediate structures with varying degrees of cation and anion disorder. **(A)** The ideal fluorite structure with a single cation. **(B)** An intermediate disordered fluorite structure with two randomly distributed cation species. **(C)** Other possible intermediates, including the disordered defect-fluorite, where both cations and vacancies are disordered and the structure is anion-deficient, and the weberite-type structures with full cation and anion ordering that differs from the **(D)** ideal ordered pyrochlore structure, which has two ordered cation species and ordered structural vacancies (showing 1/8 of the unit cell). Reproduced from (Stanek, 2003; Cleave, 2006; Zhao et al., 2017).

The pyrochlore structure can accommodate various of chemical substitutions, with over 500 unique compositions synthesized to date (Subramanian et al., 1983; Chakoumakos, 1984; Lang et al., 2010). Commonly, the A-site is occupied by a larger trivalent cation and the B-site, by a smaller tetravalent cation, with other variations (e.g. A^2+^,B^5+^) also possible (Subramanian et al., 1983). Pyrochlores can be thought of as ordered, oxygen-deficient fluorite superstructures, where the A^3+^ cations substitute half the B^4+^ fluorite cations and charge neutrality is maintained through the formation of oxygen vacancies on the 8(a) site (Chroneos et al., 2013). Both the cations and anions of pyrochlores are fully ordered on their respective sublattices, where A and B cations alternate along <110>. In addition to the fully-ordered pyrochlore and the disordered fluorite, there is a large library of partially-ordered pyrochlore and partially-disordered defect-fluorite structures (Mullens et al., 2021) (Figure 1) that vary in the degree of cation and/or vacancy ordering.

Disorder in these structures can be manipulated by tuning the cation ratio (r_A_/r_B_), which determines the stability range for different structures of the A_2_B_2_O_7_ composition (Kaspar et al., 2017), the doping content (Glerup et al., 2001), or can be introduced by the application of external stimuli (e.g., irradiation, pressure (Rittman et al., 2017a), temperature, or electric field) that can be used to replicate various types of extreme environments. Sufficient cation exchange and randomization of oxygen vacancy position in pyrochlores eventually leads to a long-range transformation to the fully-disordered, anion-deficient fluorite structure in the so-called order-disorder transition (ODT). Exposure to extreme conditions can also cause reconstructive phase transformations that result in a drastic change of structural symmetry elements (Talanov and Talanov, 2021), decomposition into other phases (Garg et al., 2008), or complete amorphization (Lian et al., 2002; Turner et al., 2017), all of which can occur homogeneously by modifying the long-range order, or heterogeneously by the formation of short- or medium-range domains of order/disorder (Shamblin et al., 2016; O’Quinn et al., 2020).

### Pyrochlore Disorder on Various Length Scales

Techniques that offer multi-scale structural and chemical characterization are necessary in disorder-harboring systems like pyrochlore and fluorite since their disordering processes occur over various spatial extents. The presence of multiple cations and structural oxygen vacancies endow pyrochlores with two types of intrinsic disorder, cation-antisite and anion-Frenkel defects (Minervini et al., 2000), which play a critical role in driving phase transitions (Subramanian et al., 1983; Minervini et al., 2000; Solomon et al., 2016; Maram et al., 2018) as well as controlling the energetics of ion transport. Randomization of cation species is a low-energy disordering mechanism in pyrochlores that arises via the formation of antisite defects ( $A_{A}^{x}+B_{B}^{x}\to \;A_{B}^{'}+\;B_{A}^{\cdot }\;$ ) and is always accompanied by simultaneous vacancy disordering in the anion sublattice (Wuensch and Eberman, 2000) (Talanov and Talanov, 2021), which occurs by the formation of anion-Frenkel pairs ( $O_{O}^{x}\to \;V_{O\left(48f\right)}^{..}+\;O_{i\left(8a\right)}^{''}$ ).

Crystalline defects (e.g., dislocations, homo- and hetero-interfaces) and other extended structural irregularities such as strain fields are shown to control cation anti-site and anion-Frenkel defect concentrations, and can also enhance pyrochlore phase-transformation kinetics under applied pressure (Rittman et al., 2017b) and irradiation in fluorites (Schuster et al., 2009). Strain-induced structural distortions such as misfit dislocations, cation intermixing, and oxygen vacancies (Bowman et al., 2015; Bowman et al., 2017; Perriot et al., 2017; Tong et al., 2020) commonly arise at interfaces and can lead to unique chemical, transport and radiation responses (Haider et al., 1998; Gázquez et al., 2017; Lehmann et al., 2017; O’Quinn et al., 2017; Shamblin et al., 2018; Gussev et al., 2020). Heterointerface engineering of fluorites, perovskites and pyrochlores has been shown to control local oxygen diffusivity (Schweiger et al., 2017), to affect local radiation response (Spurgeon et al., 2020) and to produce novel topological phases (Gallagher et al., 2016; Li et al., 2021). Other types of extended crystalline defects, such as dislocations (Sheth et al., 2016; Shafieizadeh et al., 2018) and grain boundaries (Bowman et al., 2015; Bowman et al., 2017; Perriot et al., 2017; Gupta et al., 2020; Syed et al., 2020; Tong et al., 2020) have been shown to behave as fast oxygen transport pathways in some cases. Even small, localized, atomic displacements such as local changes in bonding environment (e.g., coordination), polyhedral distortions (e.g., bond length or angle), polyhedral tilting and the adoption of short-range polyhedral configurations or ‘structural motifs’ (Shamblin et al., 2016; Sun et al., 2016) can affect functional properties. Needless to say, characterization over all these length scales is required.

In general, complex oxides are challenging systems in which subtle changes in structure or chemistry may result in colossal changes in macroscopic physical behavior (Gázquez et al., 2017). The (S)TEM techniques that will be presented here are able to directly probe the multi-scale defects presented above as well as locally ordered/disordered domains that occur in these systems, both of which may manifest on sub-unit-cell or few-unit-cell length scales. In contrast to X-ray or neutron diffraction, two structural characterization methods that yield information averaged over large volumes (1–10 μm^3^) (Lehmann et al., 2017), (S)TEM optics can be adjusted to form probes ranging from the angstrom to micrometer scale, allowing materials to be analyzed on the relevant length scales. This is critical for understanding heterogeneous disordering processes with different short- and long-range structural motifs, for example the formation of sub-nanometer domains of tetragonal symmetry in spinels (O’Quinn et al., 2017) and orthorhombic, or Weberite domains in defect-fluorites (Shamblin et al., 2018) that may be characterized by distinct space groups. Accurate structural analysis of metal oxide systems also requires sensitivity to light elements in the presence of heavy ones. For instance, the poor sensitivity of X-ray diffraction to oxygen in the presence of Y and Ta results in different space group classifications (*C222*_*1*_ and *Ccmm*) for the same Weberite-type structure, where the only significant difference is within the local atomic arrangement of the anion sublattice (Gussev et al., 2020). While this challenge still exists in (S)TEM, electrons interact much more strongly with matter than either neutrons or X-rays and the sensitivity to light elements that can be tuned by controlling the range of electron scatter collected.

Here, we review modern and emerging transmission electron microscopy (TEM) and scanning TEM (STEM) techniques that possess adequate spatial resolution and sensitivity to analyze important types of multi-scale defects, disorder and locally-ordered domains that occurs pyrochlore, fluorite, and disordered crystalline structures in general. We hope to provide exposure to these techniques and demonstrate how they may address contemporary challenges of disordered crystal characterization, aided by examples of their application in relevant systems. We also draw attention to recently developed (S)TEM techniques that have yet to be applied to pyrochlore and fluorite systems in hopes of inspiring future research efforts.

## (S)TEM and Its Capabilities

Over the past ∼20 years, sub-angstrom point-resolutions have become readily achievable in (S)TEM instruments, largely due to spherical (Haider et al., 1998) and chromatic aberration-correction (Batson et al., 2002) of electron lenses, and bolstered by improved electron sources as well as mechanical and electrical stability (Zhu and Dürr, 2015). Simultaneously, improvements in electron (MacLaren et al., 2020) and spectroscopic (Nylese and Rafaelsen, 2017; D’Alfonso et al., 2010) signal detection technologies have gradually enabled more rapid, and high signal-to-noise data collection (Hart et al., 2017). Paired with intelligent software design, multiple kinds of signals can be correlated to produce rich, multidimensional data cubes that can be flexibly analyzed. These technologies have popularized techniques that were once practically limited by the sluggish rate of signal collection, such as four-dimensional (4D)-STEM, and spectrum-imaging (SI) that are used for two-dimensional (2D) mapping of structural and chemical features, respectively. Meanwhile, (S)TEM sample holder technology also advanced, enabling dynamic, in-column experimentation whereby various stimuli can be applied to a material while functional properties are measured (Taheri et al., 2016). These *in situ* or *in operando* experiments also benefit greatly from the development of new, fast detectors which are better equipped to observe dynamic processes. Thanks to these developments and others, many of the technical barriers have been removed (MacLaren and Ramasse, 2014) such that atomic-scale structural and chemical (S)TEM analysis of materials is broadly accessible.

### Signals and Detection in the (S)TEM

**FIGURE 2:**
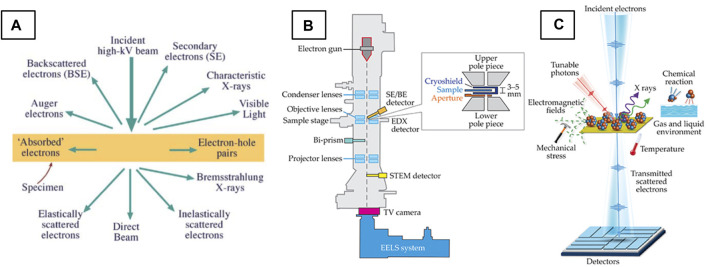
Signals and detectors of the (S)TEM. **(A)** Schematic of signals produced through electron-matter interactions in the (S)TEM (Williams and Carter, 2009a) and **(B)** schematic of (S)TEM column hardware and detectors. **(C)** Schematic of *in situ/operando* capabilities (Zhu and Dürr, 2015).

High-energy electrons (typically 80–300 kV) interact strongly with matter, producing a multitude of signals (Figure 2A) which carry valuable information about the specimen. (S)TEM imaging utilizes elastically and inelastically scattered electrons which interact with and pass through a thinned specimen, ideally below 100 nm in thickness. Secondary signals such as Auger, backscattered and secondary electrons, characteristic and Bremsstrahlung X-rays, as well as visible, infrared and ultraviolet light are also produced (Williams and Carter, 2009a) in this interaction. With the appropriate detectors (Figure 2B), multiple of these signals can be collected simultaneously. Characteristic X-rays and inelastically scattered electrons can be collected to perform energy-dispersive X-ray spectroscopy (EDXS) and electron energy-loss spectroscopy (EELS), respectively, two spectroscopic techniques central to (S)TEM that are out of the scope of this review. Specialized holders can also be used to apply different stimuli to the sample (Figure 2C) and detect the material’s functional response (see (Taheri et al., 2016) for a review of holder functionality). The diversity of these signals and the ability to detect them with high spatial resolution underpins the (S)TEM’s unique and versatile characterization power.

### Operational Modes

(S)TEMs are sophisticated optical instruments that allow control over the angular range of electron beam incidence (i.e., the convergence angle, *α*), the size of the electron beam (i.e., probed volume), and the angle of electron scatter collected (i.e., collection angle, *β*) post-specimen. It is this control, paired with various detector types and geometries, that gives rise to the broad range of (S)TEM techniques available. (S)TEM optics can be configured to operate in two main modes. In “TEM mode” (also called conventional TEM or CTEM) a broad, parallel electron beam with a flat wavefront of constant phase illuminates the specimen. Under parallel illumination such as this, the beam is typically broad and diffracted signals are localized to spots in the diffraction plane. In “STEM mode,” the electron beam is converged to a point and scanned across the specimen while the scattered signal is collected pixelwise by detectors of various geometries (Figure 4, see STEM Imaging Modes). Under convergent illumination, the incident electron wavefront is spherical, and the beam contains electrons which illuminate the specimen over a range of incident angles, from normal incidence (0 mrad) up to the convergence angle (∼15–30 mrad) (Williams and Carter, 2009b). The range of incident angles will delocalize the diffracted signal and can give access to additional crystallographic information in the diffraction plane (see Convergent-Beam Electron Diffraction). There are also special, quasi-parallel illumination conditions (see Small-Beam Electron Diffraction) that strike a balance between the typical CTEM and STEM conditions, resulting in an electron probe with near-parallel illumination and relatively small probe sizes (from several angstroms to nanometers). These optical conditions provide the basis of 4D-STEM measurements, discussed in a later section.

## Structure Analysis From Real-Space Imaging

Analysis of structure can be performed either from real-space data (images), through observation and quantification of atomic position and signal intensity, or from reciprocal-space data (diffraction patterns), via the acquisition of electron diffraction (ED) patterns of various kinds. In this section, we discuss real-space structure analysis performed in both CTEM and STEM modes and provide examples of analysis on such images to reveal local structure and the various disorders discussed previously.

### Conventional TEM

CTEM techniques utilize a broad, parallel beam to illuminate the specimen. The main real-space structure imaging approaches performed in this mode are bright-field TEM (BF-TEM), dark-field TEM (DF-TEM), weak-beam dark-field (WBDF), and high-resolution TEM (HRTEM). BF-/DF-/WBDF- TEM are primarily for morphological and defect analysis and HRTEM gives atomic-resolution images of the lattice, so it is the method of choice for analyzing atomic-scale disordering in CTEM.

#### High-Resolution TEM

HRTEM (Fultz and Howe, 2008) is a parallel imaging technique for atomic-resolution analysis of crystallographic structural information. The contrast of HRTEM images arises from the interference of electron waves as they are phase-shifted by their interaction with the interatomic potential of the specimen, generating ‘phase contrast’ (Williams and Carter, 2009c). Under the proper conditions, cation columns in a metal-oxide can be directly imaged and peak-fitting routines (see Real-Space Structure Analysis Software Tools) can be used extract cation positions with picometer precision (Lawrence et al., 2021).

The primary challenge of HRTEM imaging is that it is only representative of the projected crystal structure, or directly interpretable, under specific conditions so image simulations are typically required for accurate image contrast interpretation (MacLaren and Ramasse, 2014). For instance, in an uncorrected TEM, where the spherical aberration coefficient, C_s_, of the objective or image-forming lens is always positive, images taken at the special ‘Scherzer defocus’ condition (Scherzer, 1949) will show atomic columns as dark regions (dark atom contrast). However, if the focus, specimen mass-thickness, or crystallographic tilt changes, the resulting image may display contrast reversals where atomic columns contrast may vary across the image, appearing either bright or dark depending on local sample conditions. Two common methods to bypass this issue are exit-wave reconstruction (EWR) and crystallographic image processing (CIP). To determine aperiodic atomic structure, such as in the disordered or defective regions of disordered crystalline systems, exit-wave reconstruction is preferred since CIP relies on structural periodicity (Thust et al., 1996).

#### Exit Wave Reconstruction

**FIGURE 3:**
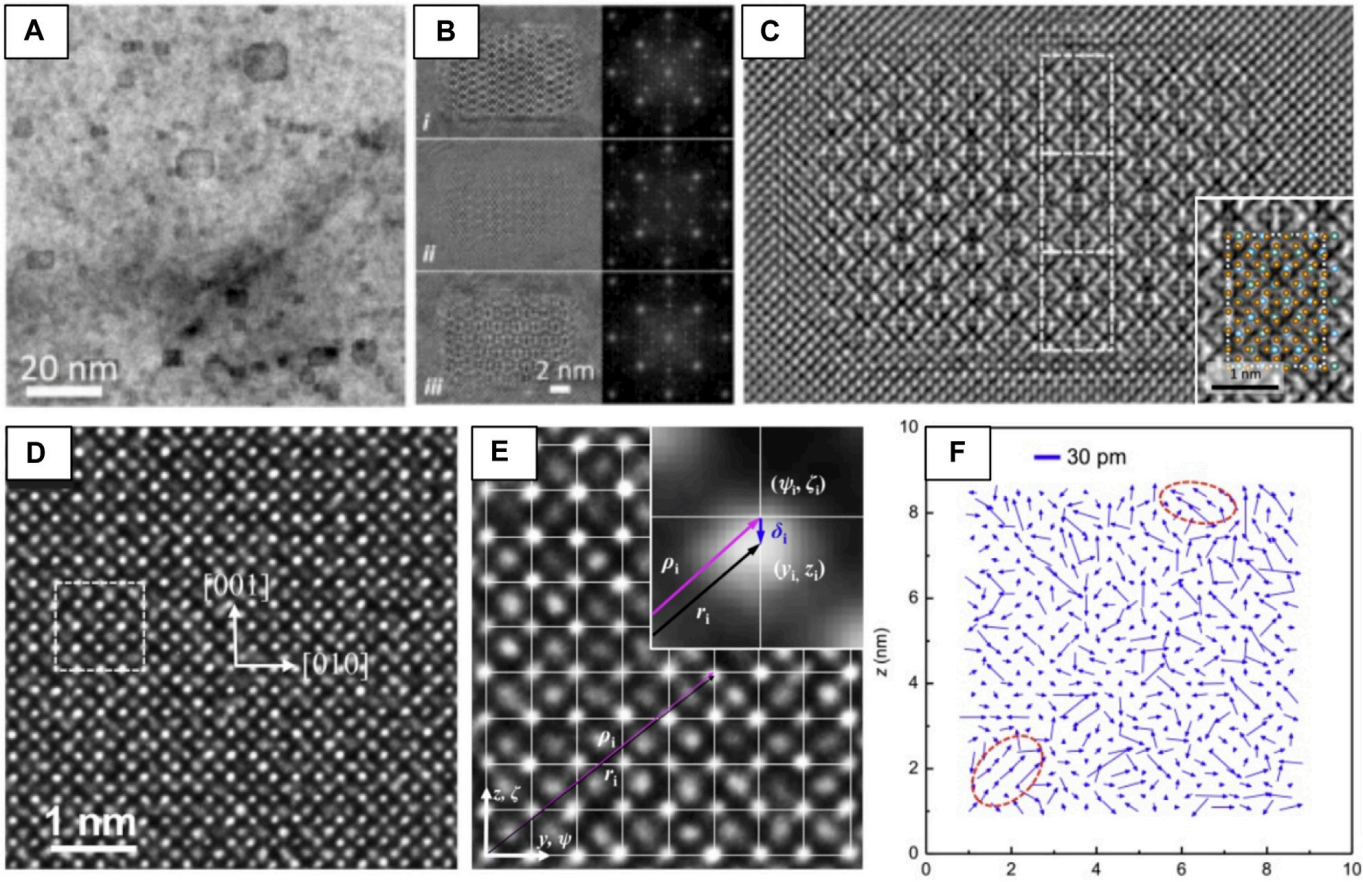
Examples of two HRTEM imaging-based techniques, EWR **(A–C)** and NCSI **(D–F)** used to determine crystal structure and atomic displacive disorder, respectively. **(A)** BF-TEM of Y_2_Ti_2_O_7_ nano-oxides in a NFA matrix. **(B)** i) underfocused, ii) focused, and iii) overfocused HRTEM images and fast-Fourier transforms (FFTs) of the nano-oxides. **(C)** Reconstructed electron wave phase obtained from EWR with white atom contrast and a simulated Y_2_Ti_2_O_7_ structure overlaid on a magnified view of the EWR (inset), reproduced from (Wu et al., 2016). **(D)** HRTEM of a [ $\bar{1}$ 00] BZN pyrochlore taken under NCSI conditions, dotted lines outline a unit cell. **(E)** Determination of the atomic displacement vector δ_i_ from NCSI images, white line crossings indicate the average cation positions of the ideal pyrochlore. **(F)** Map of shift vectors between the center of gravity of the A-site and the ideal A-site cation position, red circles outline the presence of nanoregions (∼2 nm) of correlated A-type atomic shifts, reproduced from (Jia et al., 2018).

EWR can be used to reconstruct a representative image of the projected crystal structure through the acquisition of a ‘focal-series,’ or a stack of HRTEM images taken over a range of focus values. EWR has been used to discern the structure, orientation relationship, and matrix coherency of Y_2_Ti_2_O_7_ pyrochlore nano-oxides in a nanostructured ferritic alloy (NFA) (Figure 3A). Figure 3B demonstrates the complexity of determining structure from HRTEM images, showing the difference in apparent structure in underfocused, focused, and overfocused (Figures 3Bi–iii) conditions. After acquisition of a focal series, the reconstructed phase image matches with a Y_2_Ti_2_O_7_ pyrochlore structure simulated under compressive strain (Figure 3C) (Wu et al., 2016).

#### Negative Cs Imaging

An additional challenge of HRTEM arises due to the difference in scattering power between light and heavy elements. In the case of pyrochlores (and metal oxides in general), the phase-contrast generated from cations is typically strong due to their high nuclear charge, while oxygen generates poor phase contrast and has a relatively low scattering power (Jia et al., 2003) which can make imaging of the oxygen sublattice challenging. With the development of aberration-correctors, it is possible to precisely tune the optical parameters of the (S)TEM (MacLaren and Ramasse, 2014). When the spherical aberration coefficient (Cs) is set to a small negative value, ‘negative Cs imaging,’ (NCSI) (Jia et al., 2003; Jia et al., 2004) can be performed. This method delivers directly interpretable images where both heavy and light elements show strong bright-atom contrast simultaneously (Dunin-Borkowski et al., 2016). Figures 3D–F shows a HRTEM image of a Bi-containing pyrochlore (Bi_1.5_ZnNb_1.5_O_7_, BZN) acquired under NCSI conditions, where cation columns are bright and well-defined and oxygen columns are bright, yet diffuse. 2D Gaussian peak-fitting is performed to determine precise atomic positions and displacements from ideal pyrochlore lattice positions, providing direct evidence of atomic-scale displacive disordering on the A and O′ sites (Levin et al., 2002; Jia et al., 2018). In this case, EWR and NCSI imaging **are** combined to optimize the bright atom contrast condition (Wu et al., 2016).

Although HRTEM imaging is being progressively replaced with scanning TEM (STEM) techniques due to the relative ease of image interpretation, there remains a clear advantage to using a static, broad electron beam for dynamic experiments over a scanned electron probe. Rapid specimen evolution during *in situ* experiments necessitates high temporal resolution image acquisition. In TEM mode, the acquisition rate is mainly limited by the desired signal-to-noise ratio and the frame rate of the signal detection system. However, the STEM acquisition rate is additionally limited by scan rate of the probe. For modern systems, this is on the order of a few frames per second (fps) (Ishikawa et al., 2020), where faster scan speeds can lead to significant image distortions (Sang et al., 2016). Although newly developed probe-scanning systems have demonstrated acquisitions of 25 fps (512 × 512 pixels) in STEM, approaching the necessary frame rate for many *in situ* experiments (Levin, 2021), TEM mode is still presently the choice for *in situ* experiments due the high frame rates of modern CMOS cameras (e.g., 1,600 fps at 512 × 512, K3 Gatan Inc.). *In situ* TEM experiments are routinely performed at atomic resolution, for example to track picoscale atomic surface rearrangements of CeO_2_ catalytic nanoparticles imaged under low-dose NCSI imaging conditions with millisecond temporal resolution (Levin et al., 2020a).

### Scanning TEM

In “STEM mode”, the electron beam is converged to form a probe and scanned across the specimen while various STEM detectors are positioned beneath the specimen that register the intensity of scatter at each probe position. The angle of electron scatter collected (β) is the basis for image interpretation, thus, considering its significance the various STEM imaging modes are named in reference to this collection angle: High-angle annular dark-field (HAADF), annular dark-field (ADF), low or medium angle dark-field (LAADF/MAADF), annular bright-field (ABF), or bright-field (BF) STEM. However, the angle of electron scatter is also affected by the probe convergence angle (α), so both α and β should be considered to acquire images in any of the modes mentioned above. These modes imply a specific scattering mechanism is dominant and provide physical meaning to the image contrast observed.

**FIGURE 4:**
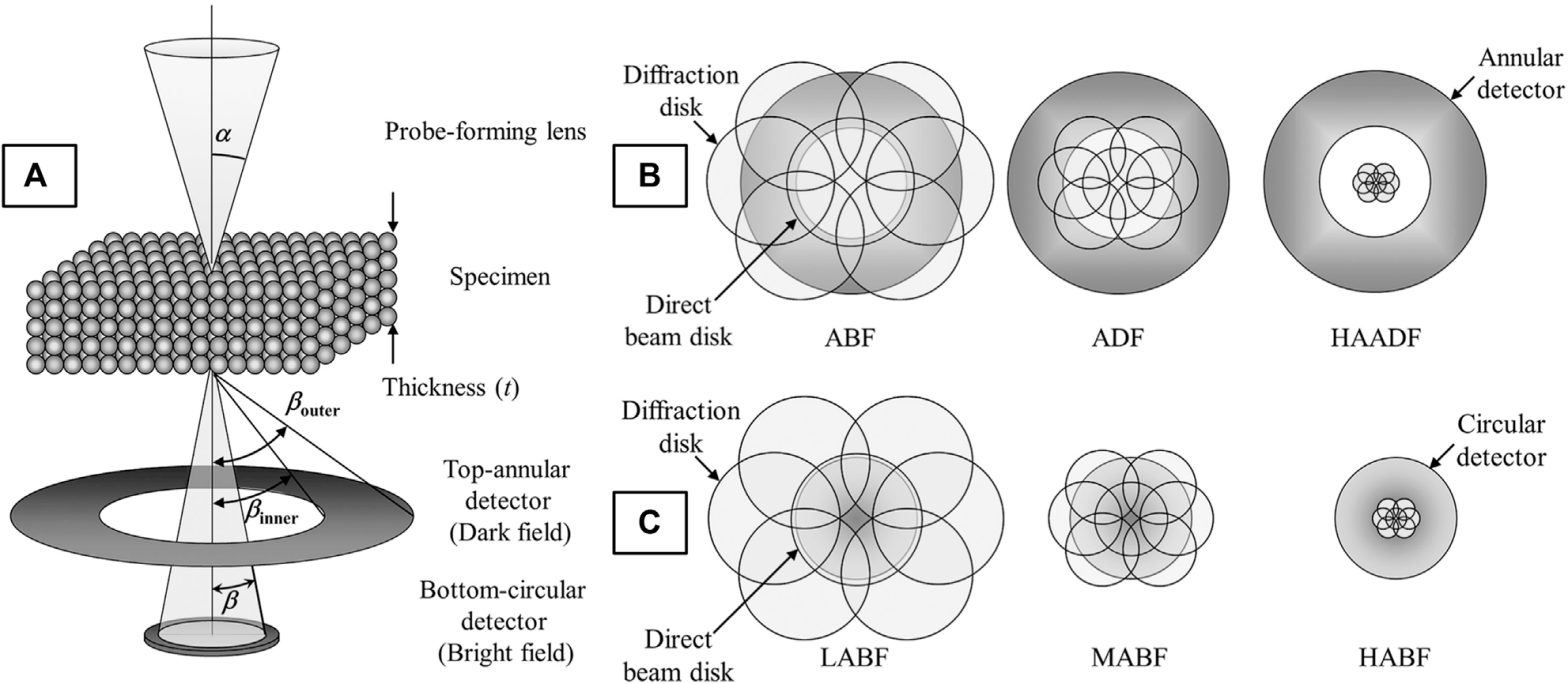
Schematic of STEM detection configuration for **(A)** double-detector STEM imaging, **(B)** annular DF (ADF) detector under ABF, ADF, and HAADF conditions, and **(C)** a circular BF detector under low, medium, and high ABF conditions (LABF, MABF and HABF, respectively). The camera length (CL) is shortened from left to right in **(B,C)**. Reproduced from reference (Kotaka, 2012).

Probe convergence angles typically fall in the range of 15–30 mrad (15–20 mrad for uncorrected systems and 25–30 mrad for aberration-corrected systems since the probe can assume a (nearly) constant phase over a greater angular range). The angle of electron scatter collected by annular detectors is *β*_*outer*_ - *β*_*inner*_, and for circular detectors just *β* (Figure 4A). Figure 4A also shows the angles *α*, and *β*, as well as the setup of a double-detector STEM system, whereby an annular detector and circular detector can be used to collect high-angle and low-angle scatter simultaneously. This multi-signal setup can be easily paired with EDXS, or, if the circular detector is removed then the primarily inelastic, low-angle scatter, can be directed into an EELS spectrometer so chemical analysis and imaging can be performed simultaneously. Figures 4B,C demonstrate that the imaging mode (and collection angle of scatter) depends not just on the type of detector used, but physical dimensions of the detectors, their positions in the TEM column and how the signal is projected onto the detector (‘camera length’). All of these factors, in addition to the probe convergence will affect the collection angle so these values need to be considered on a per-microscope basis.

#### STEM Imaging Modes

In high-angle annular dark-field STEM (HAADF-STEM), elastic, incoherent, high-angle Rutherford scatter is collected (*β*_*inner*_ > *α*) (MacLaren and Ramasse, 2014). Thus, the image intensity is proportional to the mean value of Z^n^; where *Z* is the effective atomic number, and *n* is a constant between 1.5 and 2 (Kirkland, 1998; Nellist et al., 2007). This technique is the most widely used because the atomic positions are clear and there are no contrast reversals (as in HRTEM). However, it is not ideal for imaging light elements since the Rutherford scattering process is inefficient for light atoms with low nuclear charge at high angles. In medium- (or low-) angle annular dark-field STEM (MAADF-/LAADF-STEM), lower angle coherent scatter is collected (*β*_*inner*_ ≥ *α*) where *β*_*inner*_ is just slightly larger than *α* (e.g., for α = 15–30 mrad, *β*_*inner*_ = 30–60 mrad). These images retain some of the characteristics of HAADF images (e.g., no contrast inversions, direct interpretability of atomic columns over a large focus range) but also reveal strongly diffracting features (strained areas such as dislocations or other crystalline defects, nanosized coherent or semi-coherent precipitates etc.) (MacLaren and Ramasse, 2014).

In annular bright-field STEM (ABF-STEM) the outer section of the bright-field (BF) disc is collected (*β*_*inner*_ < *α* < *β*_*outer*_) (Hammel and Rose, 1995; Findlay et al., 2010a). In contrast to images formed by the center of the BF disc which are dominated by phase contrast and are subject to contrast reversals as in HRTEM, the outer part of the BF disc produces images that are more incoherent with dark atomic contrast, including light atoms such as oxygen (Findlay et al., 2010a) or even hydrogen (Findlay et al., 2010b). Bright-field STEM (BF-STEM) is analogous to bright-field TEM images attained in TEM, especially at very low collection angles. Here the low angle scatter is small (*β* ≤ *α*), and the signal is coherent and dominated by phase contrast. Just as in HRTEM, the contrast is critically dependent on sample thickness and microscope defocus, and can show contrast inversions. (MacLaren and Ramasse, 2014).

#### Applications of HAADF-STEM

##### Strain-Stabilized Cation Disorder

**FIGURE 5:**
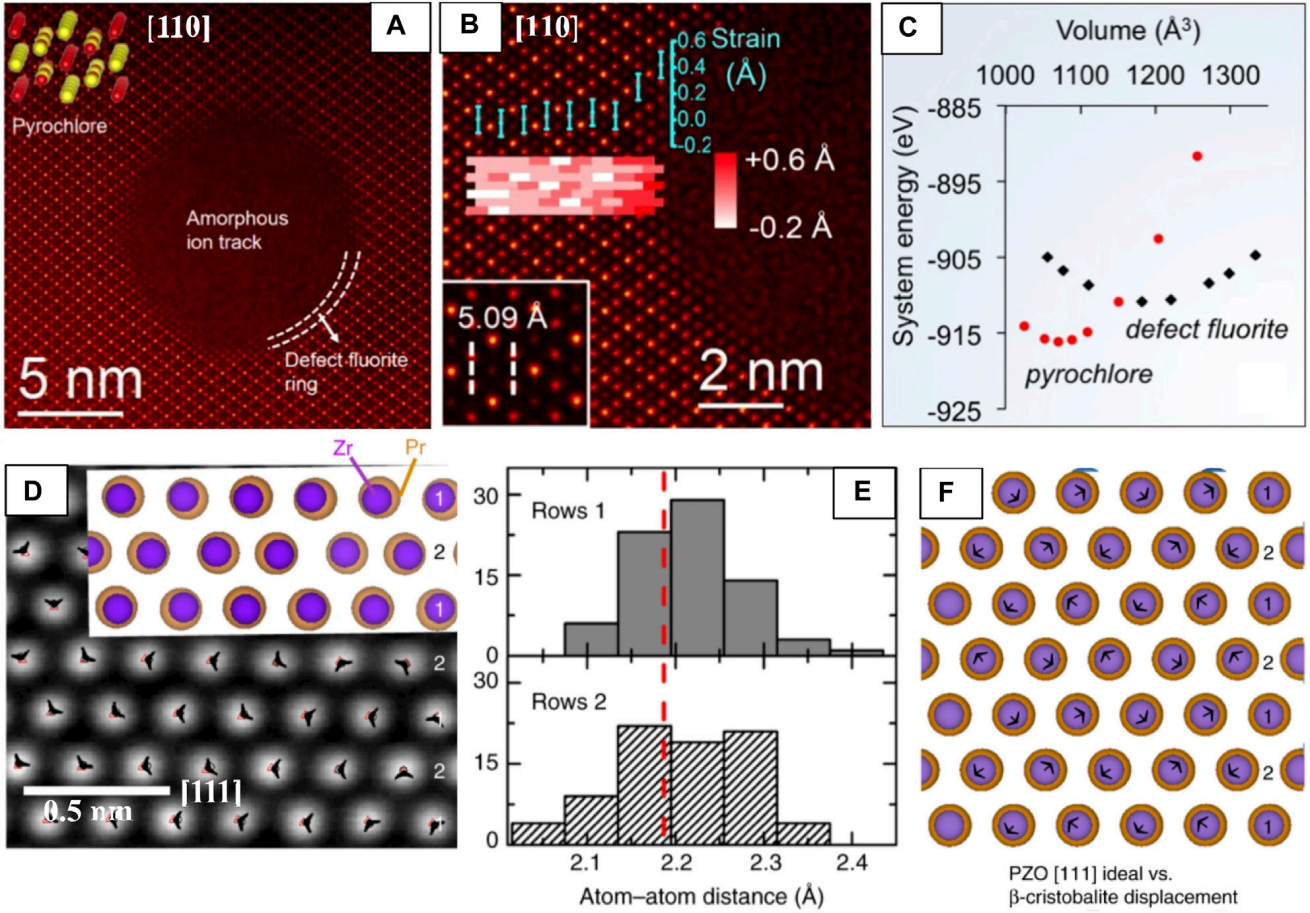
HAADF-STEM used for high-precision determination of atomic column displacements for local strain estimate **(A–C)** and for pm-scale displacive disordering in PZO **(D–F)**. **(A)** An ion track in Gd_2_Ti_2_O_7_ [110] after irradiation showing a defect-fluorite shell (dotted lines) stabilized between the amorphous core and pyrochlore matrix. An atomic model (Gd → yellow, Ti →red) (top left). **(B)** Magnified ion track edge and Gd-Ti interatomic distance of pristine Gd_2_Ti_2_O_7_ (bottom left inset) with a strain map overlaid, suggesting higher strain (bright red) approaching the amorphous core/defect-fluorite shell, error bar is ±0.1 Å **(C)** DFT energy comparison of the pyrochlore and defect-fluorite structures versus volume showing defect-fluorite has a lower system energy and greater volumes, reproduced from reference (Aidhy et al., 2015). (**D)** PZO [111] with the distorted *P*4_3_2_1_2 structure overlaid. The displacement vectors (black arrowheads) of each atomic column from the ideal ( $Fd\bar{3}m$ ) position (black circles) to experimental (red triangles) positions. **(E)** Histogram of the distance between cation columns in PZO [111] in each alternating row (1 and 2, indicated in **(D)**), red dashed line indicating the ideal ( $Fd\bar{3}m$ ) atomic column spacing. **(F)** Comparison of ideal $Fd\bar{3}m$ structure versus a *P*4_3_2_1_2 structure with an exaggerated 0.03 nm Pr β-cristobalite displacement in the [111] direction. Black arrows demonstrate displacement vectors. Displaced Pr cations shown in blue. Pr are shown in orange and Zr in purple, with Zr cations reduced by 20% for clarity. Reproduced from reference (Trump et al., 2018).

Pyrochlores and other nuclear waste storage materials are often subjected to swift heavy ion irradiation to explore how different factors (e.g., pyrochlore composition, stopping power, irradiation temperature etc. (Lang et al., 2015)) affect local structural disordering processes and thus, their overall radiation response. HAADF-STEM images of Au irradiated Gd_2_Ti_2_O_7_ shows the ion track structure consists of an amorphous core surrounded by a thin (∼1 nm thick) defect-fluorite shell, apparent from the loss of cation order as compared with the pyrochlore matrix (Figure 5A) (Lang et al., 2015). Precise calculation of atomic column positions from HAADF-STEM images shows that the structure is increasingly strained at the ion track edge approaching the core (Figure 5B). The larger interatomic distances from the HAADF image suggest that the defect-fluorite structure has a larger volume than the pyrochlore structure. Although defect-fluorite is unstable with respect to pyrochlore for the volume associated with the equilibrium pyrochlore structure, density-functional theory (DFT) calculations showed that defect-fluorite eventually becomes more stable at larger volumes (Figure 5C). Thus, these calculations show that strain could also be used to tailor oxygen conductivity in these materials, like fluorite and perovskite materials. STEM studies are being increasingly utilized for analyzing ion track morphology (Lang et al., 2015; Sachan et al., 2017a; Sachan et al., 2017b).

##### **β**-Crystobalite Disorder

Evidence of atomic displacive disorder, commonly associated with Bi-containing pyrochlores has recently been seen in other pyrochlores (e.g., Pr_2_Zr_2_O_7_ (PZO), La_2_Zr_2_O_7_, and Yb_2_Ti_2_O_7_) using HAADF-STEM. With rigorous treatment of scan-distortion correction, and peak-fitting procedures, the authors reveal static, β-cristobalite type (Seshadri, 2006) A and O’ displacements on the order of ∼0.01 nm (Figures 5D–F) which lower the local symmetry and surprisingly, can exist in the absence of cation disorder, non-stoichiometry, and lone-pair effects. The authors propose that such static displacements are common in pyrochlores and are driven by cation size mismatch rather than defect concentrations or electronic effects (Trump et al., 2018).

##### Cation Vacancy Order

**FIGURE 6:**
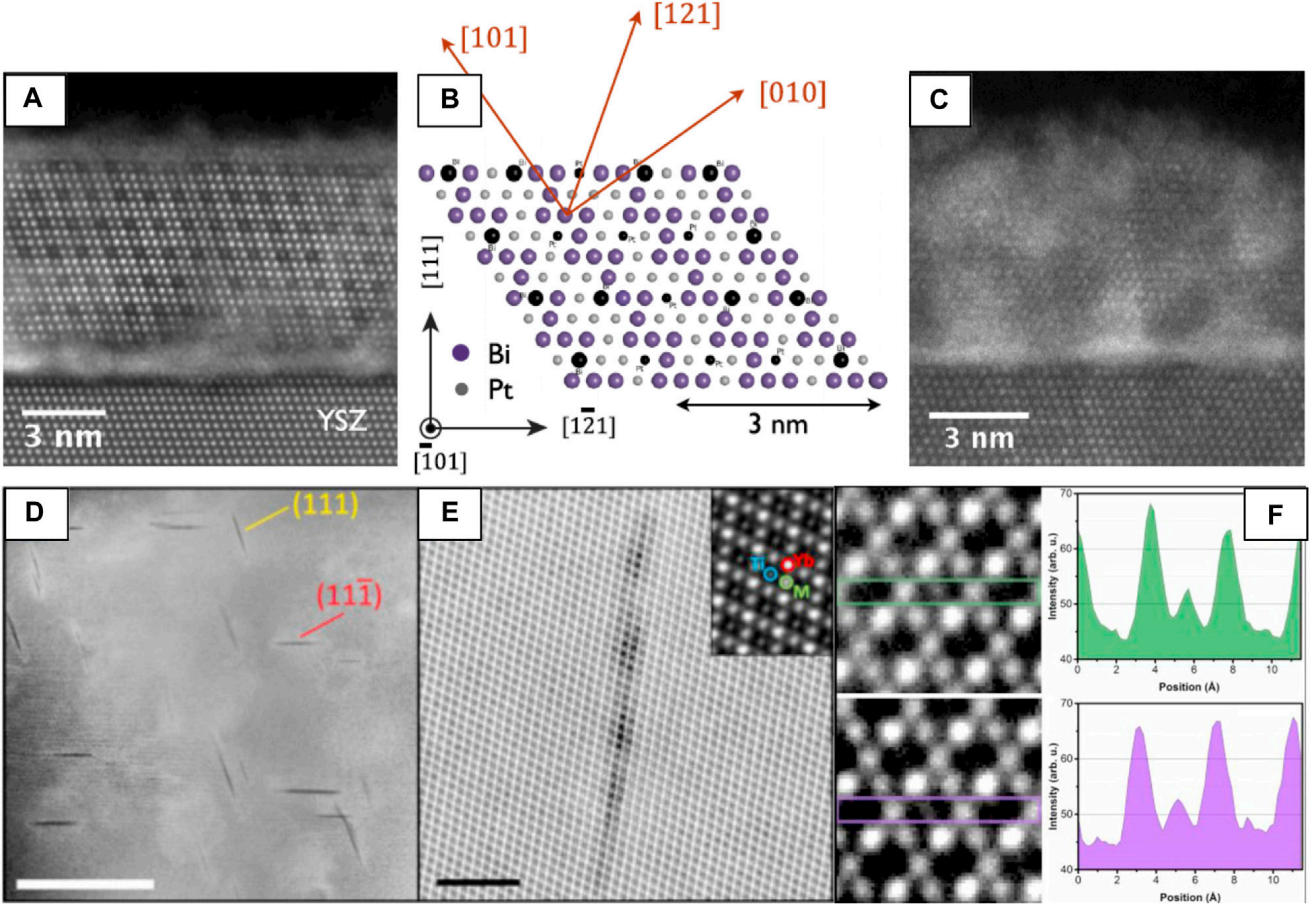
HAADF-STEM imaging showing cation **(A–C)** vacancies and (d–f) anti-site defects. (a) HAADF-STEM image of ordered Bi_2_Pt_2_O_7_ [ $\bar{1}01$ ] epitaxial pyrochlore in the annealed bismuth-rich film. Dark, ordered atomic positions in the image indicate the presence of ordered cation vacancies. **(B)** Atomic model of the Bi_2_Pt_2_O_7_ [ $\bar{1}01$ ] pyrochlore, where black positions denote cation vacancies, as seen in the image in **(A)**. **(C)** HAADF-STEM image of Bi_2_Pt_2_O_7_ [ $\bar{1}01$ ] without cation vacancies. Reproduced from reference (Gutiérrez–Llorente et al., 2015) **(D)** ADF-STEM image of planar defects in Yb_2_Ti_2.05_O_7_ [ $1\bar{1}0$ ] irradiated with 200 keV electrons. **(E)** Magnified strain-free defect. **(F)** anomalously bright Ti columns on defect edges. Reproduced from reference (Mostaed et al., 2018).

HAADF-STEM has also been used to image cation vacancy ordering (Figure 6) in a Bi_2_Pt_2_O_7_ pyrochlore (Gutiérrez–Llorente et al., 2015), a promising oxide catalyst for applications in fuel cell technology. Direct growth of Bi_2_Pt_2_O_7_ films has proved challenging, so the authors implement a novel route whereby a δ-Bi_2_O_3_ film and Pt are co-deposited and annealed to achieve epitaxial Bi_2_Pt_2_O_7_ through δ-Bi_2_O_3_. Since HAADF-STEM provides strong Z-contrast, the cation vacancy ordering is immediately apparent (Figure 6A), especially when viewed beside the ordered pyrochlore with no cation vacancies (Figure 6C).

##### Electron Beam-Induced Cation “Stuffing”

In some pyrochlores extended crystalline defects may be formed under electron irradiation (during (S)TEM observation). In Yb_2_Ti_2_O_7_, two types of defects were observed; the majority of which induced no measurable strain in the crystal, while some displayed long-range strain-fields and dislocation character. The material surrounding the strain-free defect shows clear evidence of site-swapping between the cation sublattices (Figure 6F) where occasional Ti sites have significantly brighter contrast than their neighbors, indicating the ‘stuffing’ of Yb on Ti sites (Mostaed et al., 2018).

#### Strain Analysis of Crystalline Defects, Hetero- and Homo-Interfaces

As described previously, strain-fields in pyrochlores can dramatically affect their properties, thus, analysis of strain-fields around structural defects formed in pyrochlores formed during synthesis are critical to understand (Shafieizadeh et al., 2018). Geometric phase analysis (GPA) (Hÿtch et al., 1998) and peak analysis (Bayle et al., 1994; Anthony and Granick, 2009) are two commonly used digital processing approaches that facilitate extraction of lattice parameter at high resolution, based in reciprocal- and real-space, respectively (Zhu et al., 2013). In GPA, atomic displacements are measured by calculating the ‘local’ Fourier components of the lattice fringes of a real-space image (Hytch, 1997), whereas peak-finding methods find atomic positions from the contrast maxima of images directly, often through center of mass calculations or by 2D Gaussian fitting which can be more precise (Anthony and Granick, 2009). In either case, contrast maxima in the image are identified as a periodic lattice so deviations of the real, local lattice with respect to an unstrained reference lattice can be calculated and mapped (Zhu et al., 2013). Although these methods are generally robust, precautions must be taken to minimize microscope and specimen effects that may produce inaccurate strains, especially from HRTEM images (Hÿtch and Plamann, 2001) where contrast interpretation is often less straightforward than in HAADF-STEM.

**FIGURE 7:**
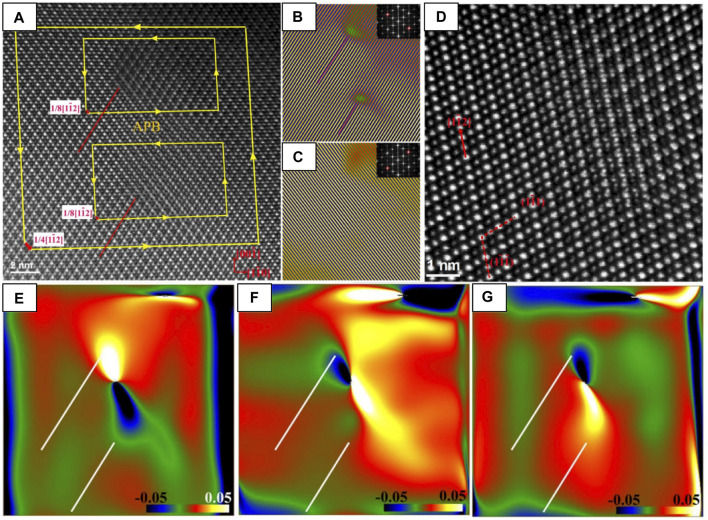
A superdislocation with Burgers vector 1/4 [112]. **(A)** The dissociated superdislocation with two partial dislocations and an APB. Three Burgers circuits are shown as yellow frames. The projected Burgers vectors are shown by the red arrows. **(B)** Inverse FFT image using **g** = ± (222); inset: FFT diffraction with **g** circled. **(C)** Inverse FFT image using **g** = ± (222); inset: FFT diffraction with g circled. **(D)** An enlarged HAADF STEM image of a 1/8 [112] partial dislocation. **(E–G)** *ɛ*_*xx*_ along [110], *ɛ*_*yy*_ along [001], and *ɛ*_*xy*_ strain maps of **(A)**; the location of the two extra atomic planes of the dislocation cores indicated by lines, reproduced from (Shafieizadeh et al., 2018).

GPA has been used to map strain fields (Figure 7) around superdislocations and other extended crystalline defects in Yb_2_Ti_2_O_7_ formed during synthesis (Shafieizadeh et al., 2018). HAADF-STEM reveals extended defects such as anti-phase boundaries and dissociated superdislocations with unique core structures and anomalously large Burgers vectors (Figure 7A) compared with other oxides (Jia et al., 2005). GPA has also been used to map strain at La_2_Zr_2_O_7_(LZO)/YSZ (111) interfaces, highlighting the presence of misfit dislocations that may impact the accumulation of radiation damage at such features (Kaspar et al., 2017). This approach has also been used to demonstrate the effect of strain at $\text{Σ}3\left(11\bar{1}\right)<1\bar{1}0>$ twin boundaries in Gd_2_Ti_2_O_7_, showing a local reduction of the oxygen migration barrier compared with the bulk (Gupta et al., 2020).

#### Applications of ABF-STEM

##### Layer Termination at Heterointerfaces

**FIGURE 8:**
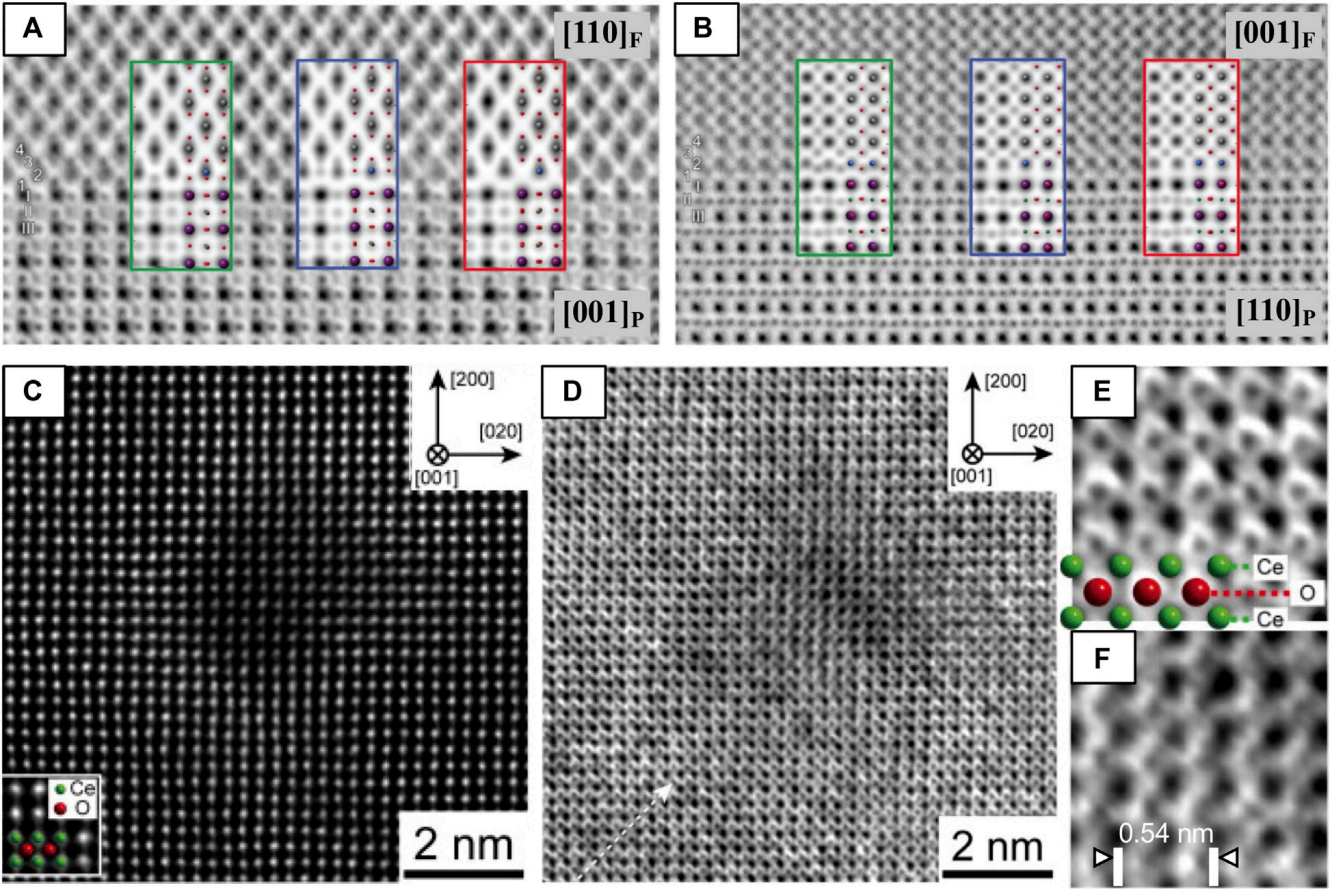
HAADF and ABF-STEM images used to **(A,B)** clarify surface termination layer at an LAO-LZO interface and to **(C–F)** investigate the oxygen sublattice of CeO_2_ after swift heavy ion irradiation. ABF-STEM images of the LaAlO_3_/La_0.5_Zr_0.5_O_1.75_ interface along the **(A)** [001]P and **(B)** [110]P zone axes. Three models of the fluorite/LaAlO_3_ interface and their simulated ABF-STEM images were compared with experimental images to map the cation and anion positions. Comparison of images and models in both **(A,B)** shows that the O_2_-terminated (green) model matches best along the two zone axes, with each cation/anion accounted for by the model. Reproduced from reference (O'Sullivan et al., 2016). **(C)** HAADF-STEM and **(D)** ABF-STEM images of an ion track in CeO_2_ [001] formed by Xe ion irradiation, with magnified views of the **(E)** peripheral and **(F)** core damage regions of the ion track. Atomic models of CeO_2_ are shown in the insets of **(C,E)**. Reproduced from reference (Takaki et al., 2016), original data from (Takaki et al., 2014).

Because of ABF-STEM’s superior sensitivity to light elements, it is the method of choice to probe the anion sublattice. In this example, it is used to map both the anion and cation positions at an LaAlO_3_/La_0.5_Zr_0.5_O_1.75_ (fluorite) interface. The authors simulated three candidate structural models of terminations of the interface to compare with two orientations ([001] and [110]) of the interface (Figures 8A,B), finding that the model matches the two ABF images best when the fluorite layer is terminated by a bulk fluorite oxide anion layer followed by a pure Zr layer (green). This example demonstrates yet another examples of complementary nature of (S)TEM data and (S)TEM simulation (O'Sullivan et al., 2016).

##### Anion Disorder in Ion Tracks

The ability of STEM imaging to simultaneously collect multiple angles of electron scatter can be tremendously helpful for understanding the local structure. For instance, simultaneously acquired HAADF- and ABF-STEM images of ion tracks in ceria (Figures 8C–F) show that 1) a decrease in atomic density occurs inside the ion track, evident from the local drop of signal intensity in that HAADF-STEM images of the Ce lattice (Figure 8C) and 2) the O anion lattice is preferentially disordered by the electronic excitation damage at the core of the ion track, evident from blurring/absence of O anion columns at the core region from ABF-STEM images (Figures 8D–F) (Takaki et al., 2014).

STEM techniques have been used to explore the structure of various pyrochlore and fluorite systems (Gallagher et al., 2016; Sachan et al., 2017b; Sachan et al., 2018; Sachan et al., 2019; Spurgeon et al., 2019; Lee et al., 2020; Spurgeon, 2020). See reference (MacLaren and Ramasse, 2014) for a review of STEM techniques applied to functional oxides beyond pyrochlore and fluorite systems.

#### Real-Space Structure Analysis Software Tools

In addition to experimental factors, such as high-quality TEM specimen preparation, and proper optical setup and image acquisition parameters, software tools can be used to correct scan distortions of STEM images and automated procedures can perform highly accurate assessment of atomic positions. There are many software programs available to do this (e.g., Ranger (Jones and Nellist, 2013), qHAADF (Galindo et al., 2007), iMtools, StatSTEM (De Backer et al., 2016), and oxygen octahedra picker [110], as well as Atomap [111], among others [112] (Jones et al., 2015)). This is still a highly active area of research, where machine learning tools are being developed to improve image quality and atom detection (Lin et al., 2021). Image simulation is useful to aid the interpretation and validation of STEM data, as conveyed through multiple examples above. The two most commonly used image simulation methods are the Bloch wave and multislice methods, see (Kirkland, 1998) for detailed descriptions. Some popular software options for STEM image simulation are Dr. Probe (Barthel, 2018) Prismatic (Ophus, 2017; Rangel DaCosta et al., 2021), and pyQSTEM (Koch, 2002), among many others.

## Structure Analysis From Reciprocal-Space (Diffraction) Data

**FIGURE 9:**
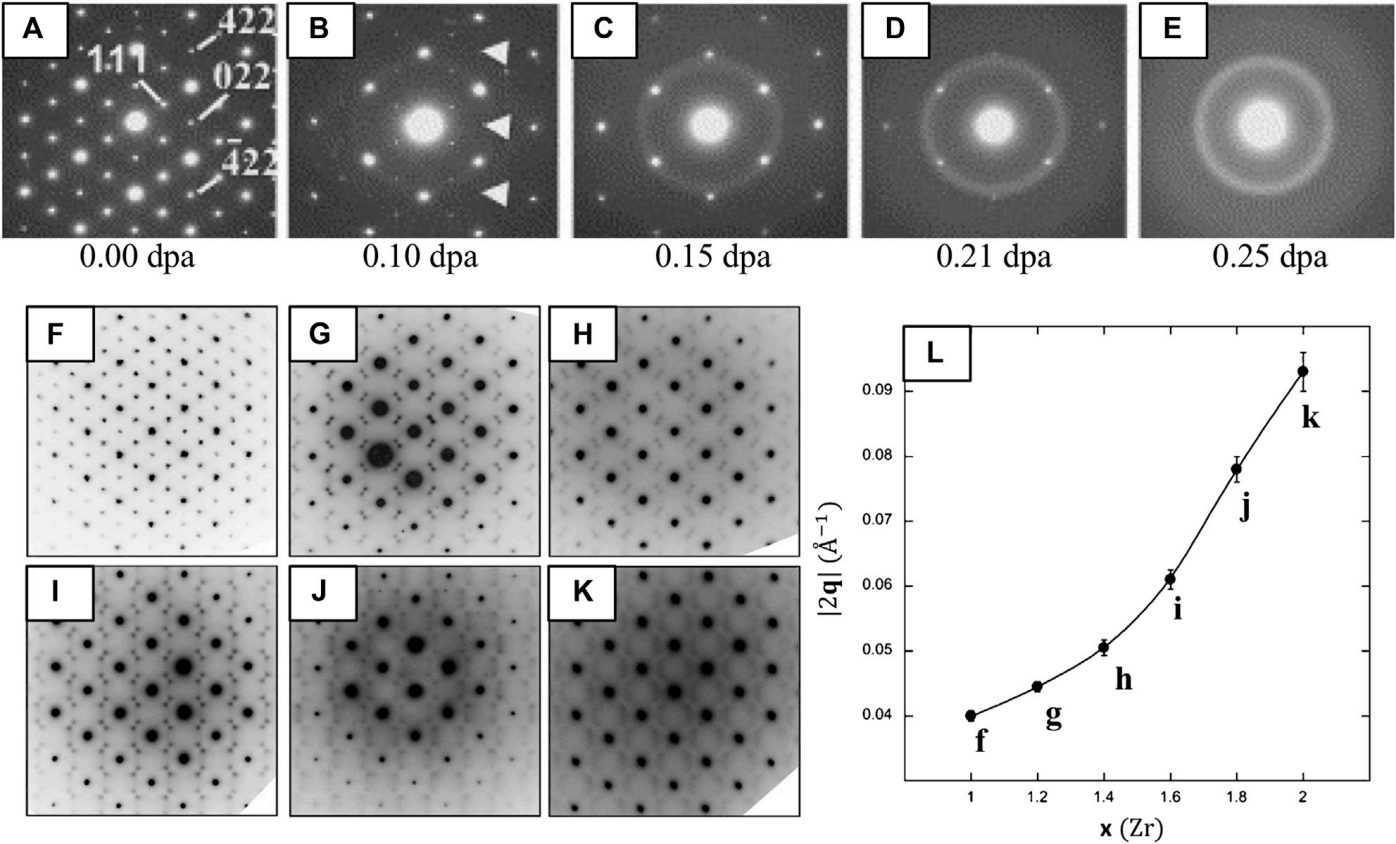
SAED patterns illustrating ODT of ordered pyrochlores **(A–E)** due to ion-irradiation and **(F–L)** chemical doping. **(A–E)** SAED diffraction patterns of Er_2_Ti_2_O_7_ [ $01\bar{1}$ ] subjected to Kr^+^ irradiation at increasing dose. The loss of diffraction maxima with indices *h* + *k + l =* 4*n* (i.e., 022 and 422) (white arrows) suggests anion disordering occurs upon irradiation, followed by cation disordering and loss of long-range order with increasing dose, reproduced from (Lian et al., 2003). **(F–L)** SAED patterns of Y_2_Sn_2−*x*_Zr_*x*_O_7_ [110] for increasing Zr content (*x*) where **(F)** *x* = 1.0 (Y_2_Sn_1.0_Zr_1.0_O_7_) **(G)** *x* = 1.2 **(H)** *x* = 1.4 **(i)** *x* = 1.6 **(J)** *x* = 1.8 and **(K)** *x* = 2.0 (Y_2_Zr_2_O_7_). **(L)** A plot of ∣2**q**∣ versus Zr content, where ∣2**q**∣ represents the characteristic spacing between pairs of satellite reflections. The real-space correlation distances for the minimum (*x* = 1.0, ∣2**q**∣^−1^ = 11 ± 0.8 Å) and maximum (*x* = 2.0, ∣2**q**∣^−1^ = 3 ± 0.2 Å) Zr contents. Reproduced from reference (de los Reyes et al., 2013).

Structural information contained in (S)TEM images is also contained in the electron diffraction (ED) pattern, represented in real-space and reciprocal-space, respectively. Electrons scattered from the specimen generate diffraction patterns that contain rich crystallographic information. From the position and symmetry of the diffraction spots, it is possible to determine the unit-cell parameters and lattice type, while the intensities of the diffraction spots are related to the arrangement of atoms within the unit cell (Jia et al., 2005). ED can also be used to assess sample crystallinity because crystalline materials produce sets of sharp and discrete Bragg reflections, whereas disordered, condensed phases such as liquids or glasses produce smooth, continuous ‘halos’ of scatter (Figures 9A–E). Selected-area electron diffraction (SAED) is the most straightforward ED method, obtained by isolating a region of the specimen for diffraction analysis with an area-limiting aperture (>0.1 μm) under parallel-illumination. However, many variations exist (see Small-Beam ED) where the (S)TEMs optical setup can be tuned to probe a smaller volume than with SAED while maintaining near-parallel convergence, which is necessary in some cases to acquire the desired information.

### Diffraction Fine-Structure

Additional structural information is encoded in the ED ‘fine structure’ (e.g., extra and split peaks, satellite reflections, and structured diffuse scattering) which provide a means to analyze defects and reveal local ordering. Fine structure can indicate the presence of superstructures (Abelson et al., 2020) of various kinds (e.g., pyrochlore cation or vacancy ordering (Lian et al., 2005)), extended defects (e.g., planar interfaces and dislocations (van Landuyt et al., 1966)), and can also signify the shape of diffracting volumes in certain situations (Edington and Edington, 1975; Williams and Carter, 2009d). Diffuse electron scatter is a type of fine structure that arises from inelastic interactions such as phonon, plasmon and electron excitations or elastic interactions with crystal imperfections (Zuo et al., 2017a). Diffuse scattering may have many different origins, for example, substitutional ordering/disordering coupled to displacive disorder as atomic positions are relaxed around vacancies or substitutional atoms of different sizes, tilts of rigid polyhedra, lattice deformation, the formation of textures and others (Van Tendeloo and Amelinckx, 1998). If the diffuse scatter can be interpreted, it is a powerful signature of local ordering over the probed volume of the specimen. However, since classical crystallography provides no established protocol for its analysis, diffuse scatter is often either ignored or analysis is carried out on a case by case basis where the tools developed cannot be broadly applied across material systems (Keen and Goodwin, 2015).

#### Applications of Diffraction Fine-Structure and Diffuse Scatter Analysis

Often, the presence of additional scattering effects (satellite and diffuse scatter) in fluorite and pyrochlore systems are a result of local, strain-driven compositional and displacive structural modulations (Withers et al., 1991; Tabira et al., 1999; Tabira et al., 2001; Withers et al., 2004; Liu et al., 2006; Withers and Hawkes, 2008; Welberry and Weber, 2016; Trump et al., 2018) and sometimes can be related to the formation of locally-ordered domains (García-Martín et al., 2005; Lau et al., 2008; Reid et al., 2012). For instance, the presence of local, C-type ordering in lanthanide oxides on short to intermediate length scales can give rise to ED fine structure; selective imaging of signal originating from single satellite peaks (satellite dark-field imaging) has been used to reveal the distribution of C-type ordered nanodomains on the order of ∼50–100 Å in real-space (Reid et al., 2012).

##### Ion-Irradiation Induced Order-Disorder Transition

ED is especially useful for observing the ODT of pyrochlores to defect-fluorite since the degradation of cation and anion ordering leads to the loss of pyrochlore superstructure reflections (Lian et al., 2005; Lian et al., 2003). For instance, *in situ* ion-irradiation in the TEM with the collection of SAED patterns shed light on how the ODT advances in the temporal domain (Figures 9A–E) (Lian et al., 2003). These authors show that pyrochlore first becomes anion-disordered as a result of Frenkel pair accumulation and oxygen vacancy redistribution, evidenced by the loss of superstructure reflections with indices h + k + l = 4n that correspond solely to anion ordering (e.g., 220, 422). This is followed by partial cation disordering, evidenced by loss of the 111 superstructure reflections that correspond to both cation and anion ordering (Figures 9A–E).

##### Chemical Doping-Dependent Correlation Length

In addition to revealing cation and anion disordering under irradiation, ED fine structure and diffuse scatter can also reveal short to medium range ordering. de los Reyes et al. (2013) use these features to examine the ODT in Y_2_Sn_2−*x*_Zr_*x*_O_7_ (0.0 ≤ *x* ≤ 2.0) upon chemical doping (substitution of Zr for Sn). SAED patterns of their starting composition Y_2_Sn_2−*x*_Zr_*x*_O_7_ (Figure 9F) are consistent with the pyrochlore structure, where the strongest diffraction maxima correspond to the fundamental Bragg reflections of the fluorite sub-cell with weak characteristic pyrochlore superlattice reflections also present. As Zr replaces Sn (*x* ≥ 1.2), the pyrochlore ordering reflections disappear and the fundamental Bragg reflections of the fluorite structure (G_F_) are decorated by pairs of satellite reflections mirroring the G_F_ ± ½ <111>^*^ regions of reciprocal-space for small *x* (*x* = 1.2–1.6) (Figures 9G–I). Note that the diffuse scattering at any position in reciprocal-space can be defined by a wave-vector **G**_F_ ± **q**, where **G**_F_ is a reciprocal lattice vector of the underlying fluorite-type cell and **q** is a modulation vector (García-Martín et al., 2005).

At higher substitutions (*x* = 1.8–2.0) the initial satellite spots weaken and the intensity transforms into a more diffuse, wavelike pattern that corresponds to a second modulation of the underlying fluorite structure at G_F_ ± ¼ <220>^*^ (Figures 9J,K), as observed in other pyrochlore/defect-fluorite systems (Suzuki et al., 1985; Gallardo-López et al., 2001). The inverse of the modulation wave vector magnitude (∣2q∣^−1^) associated with the satellites gives a real-space correlation distance (or interaction length) (Whittle et al., 2009) that decreases from ∼25 Å (x = 1.0) to ∼11 Å (x = 2.0), equivalent to ∼2 to 1 pyrochlore unit-cells, respectively. Several underlying phenomena have been proposed to explain the presence of these features, including defect-vacancy and/or interstitial ordering, ordered-cluster arrangements, micro-domain pockets, and anti-phase domain boundaries (e.g., pyrochlore order within fluorite) (Allpress and Rossell, 1979; van Dijk et al., 1986; Tabira et al., 2001). Regardless, the diffraction fine-structure can be correlated to a real-space local-ordering phenomena, where (in this case) the interaction length appears to decrease as a function of chemical doping.

##### 3D Reconstruction of Diffuse Scatter in Reciprocal Space

**FIGURE 10:**
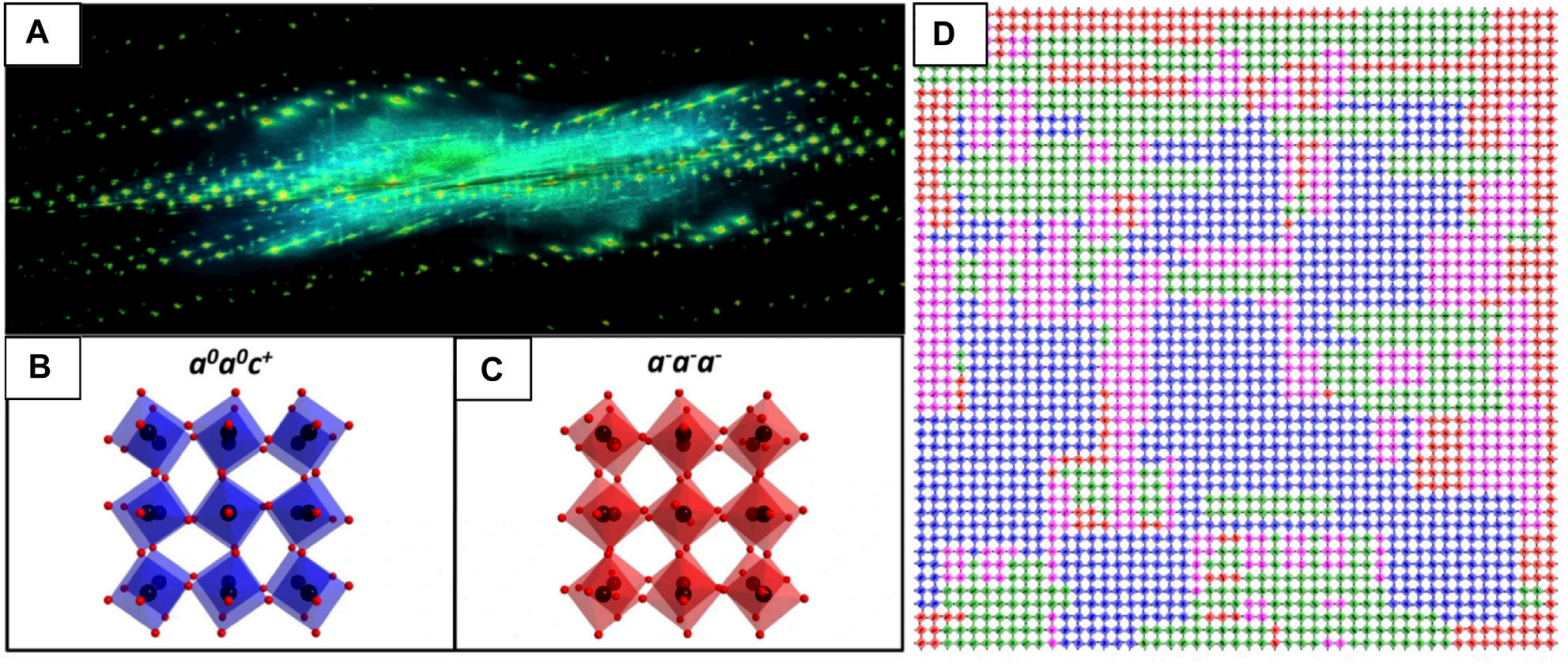
3D-ED mapping of Bragg and diffuse electron scatter in reciprocal-space to determine TiO_6_ octahedral tilt schemes in 85NBT-10BKT-5BT [001]_pc_ ceramic. **(A)** Reconstruction of the reciprocal-space volume showing intensity from both Bragg and diffuse scattering. **(B)** Schematic of the in-phase *a* ^*0*^ *a* ^*0*^ *c* ^+^ and **(C)** antiphase *a* ^−^ *a* ^−^ *a* ^−^ tilt systems. **(D)** A 2D slice cut from **(A)** showing three in-phase variants randomly distributed in the antiphase tilted matrix (red, blue, green, and magenta octahedra are tilted according to the *a* ^−^ *a* ^−^ *a* ^−^, *a* ^*0*^ *a* ^*0*^ *c* ^+^, *a* ^*0*^ *c* ^+^ *a* ^*0*^ and *c* ^+^ *a* ^*0*^ *a* ^*0*^ tilt systems, respectively. Only the TiO_6_ octahedra are displayed. Reproduced from (Neagu and Tai, 2017).

Recently, the analysis of diffuse features in electron diffraction data has extended into 3D, called 3D-ED or diffraction tomography. 3D-ED data can be acquired through various specimen tilting/rotation schemes (Gemmi et al., 2019; Kolb et al., 2007) to map the Bragg and diffuse scatter from disordered materials in reciprocal-space (Figure 10A). Rotation ED (RED) (Zhang et al., 2010; Wan et al., 2013) is one acquisition approach, which combines electron beam tilting over many small steps with crystal rotation over a few large steps. 3D-ED has not yet been utilized to explore local structural deviations in fluorites or pyrochlores, but has been used to identify and map unique oxygen octahedral tilting systems in a 85NBT-10BKT-5BT ternary piezoceramic perovskite (Neagu and Tai, 2017), among other systems. This approach can lead to much clearer interpretation of ED fine-structure, for instance the satellite reflection pairs mirroring the G_F_ ± ½ <111>^*^ regions of reciprocal-space make up a single continuous circle of diffuse intensity in 3D reciprocal-space, however this is not immediately apparent from the 2D projections shown in Figures 9F–K. Further resources are available on the interpretation (Van Tendeloo and Amelinckx, 1998; Zuo et al., 2017a) and modeling (Neder and Proffen, 2009; Keen and Goodwin, 2015) of diffuse scattering and SAED fine structure from disordered crystalline systems.

### Small-Beam Electron Diffraction - Microbeam, Nanobeam, Angstrom Beam and Convergent Beam Electron Diffraction

In SAED, the size of the electron beam on the specimen is typically >0.1 μm across and the area of the diffracting region is controlled by an area-limiting aperture. In μ-/N-/A-BED, the TEM optics are modified to achieve quasi-parallel illumination, much like SAED. However, the prefix micro, nano, or angstrom *roughly* indicate the size of the beam on the specimen, thereby limiting the size of the diffracting region. It is important to note that neither the terms μBED, NBED, ABED, CBED, nor the optical conditions they intend to describe are used consistently across the literature, so it is important to check beam size and convergence to understand the optical conditions used in any small-area diffraction study. We will refer to techniques where the probe size is minimized to provide localized diffraction information as ‘S-BED’ techniques, which is for ease of reference only and not a widely used acronym.

#### Applications of S-BED

##### Critical Dose and Temperature of Amorphization

**FIGURE 11:**
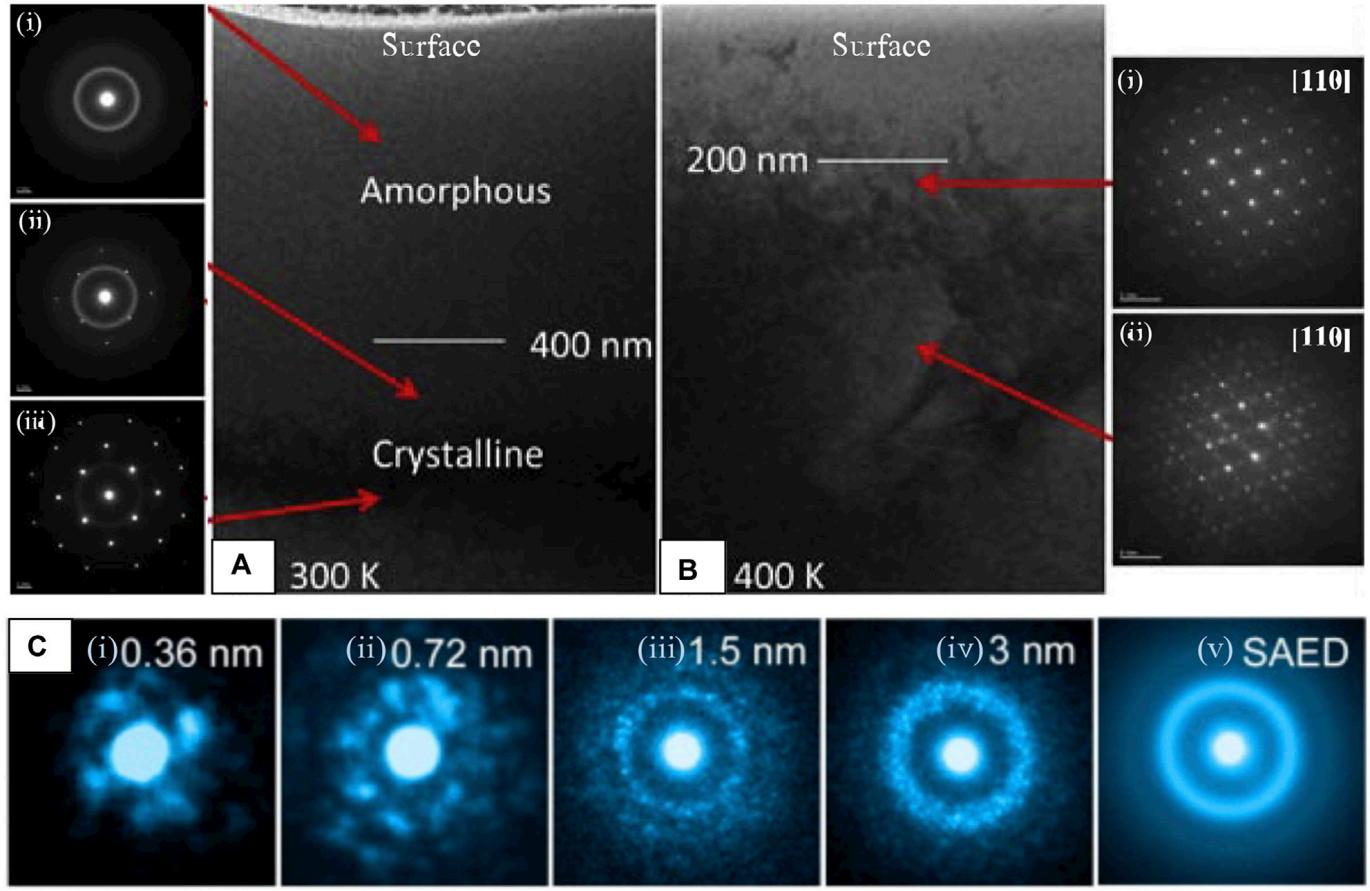
Small (nm- or Å-scale) quasi-parallel electron probes to acquire highly localized diffraction information to **(A,B)** assess crystallinity as a function of depth and **(C)** to demonstrate the effect of probe size in detection of local atomic ordering in an amorphous solid. **(A)** BF-TEM of an ion-irradiated Yb_2_TiO5 cross-section held at **(A)** 300 K, NBED showing loss of crystallinity at the **(i)** surface and **(B)** 400 K, NBED showing **(i)** absence of the (111) pyrochlore reflection which **(ii)** reappears at depth, reproduced from (Aughterson et al., 2018) **(C)** The effect of increasing probe size in a Zr_66.7_Ni_33.3_ metallic glass, where local atomic ordering emerges at the smallest (Å-scale) probe sizes, averaging out into a smooth, diffuse ring as the probe size increases. Reproduced from reference (Hirata and Chen, 2014).

S-BED techniques are particularly useful when the region of interest is limited in size, for instance, to assess damage at various depths within an irradiated Ho_x_Yb_(2-x)_TiO_5_ pyrochlore series under *ex situ* temperature-dependent irradiation (Aughterson et al., 2018). The onset dose and depth of amorphization can be precisely determined for various temperatures with NBED, which provides the information necessary to calculate the critical dose (D_c_) and temperature (T_c_) for amorphization, two quantities which indicate suitability for nuclear environments. Figures 11A,B shows a TEM cross-section of the Se^+^ irradiated Yb_2_TiO_5_ (*x* = 0) specimen at 300 K (Figure 11A) and 400 K (Figure 11B), respectively. NBED patterns acquired at increasing depths (Figures 11Ai–iii) show smooth halos of electron scatter (Figure 11Ai) in the near-surface region, indicating complete amorphization, sparse Bragg reflections (Figure 11Aii) at greater depth, indicating a mixed crystalline/amorphous state, and a fully crystalline state (Figure 11Aiii) furthest from the irradiated surface. The specimen irradiated at 400 K presented in Figure 11B provides another example of the ability to discern the state of pyrochlore ordering by the absence (Figure 11Bi) or presence (Figure 11Bii) of superstructure reflections (Aughterson et al., 2018). Determination of D_c_ and T_c_ can also be performed *in situ*, however, availability of (S)TEMs equipped with irradiation sources are limited (Hinks, 2009).

##### “Structure” of the Amorphized State

In the highly disordered regions near the sample surface of Figures 11A,B the atomic structure is either partially (Figure 11Ai) or fully amorphized (Figure 11Aii) as a result of irradiation treatment. These disordered regions likely retain some characteristic structures of their pre-irradiated parent-phase, or other defect structures that may provide clues on how the disordering process progresses. Although amorphous materials do not possess long-range order as crystals do, they often demonstrate structural correlations on the scale of a few angstroms (SRO) or nanometers (MRO) that can be teased out through various diffraction and analysis methods (Cockayne, 2007). As demonstrated by Figure 11C, new structure arises in the diffracted signal as the probe size is reduced to the nm- or Å-scale. When the probe is on the order of SRO/MRO, local symmetries of individual structural units appear as the probed volume approaches the structural unit size, providing many new analytical opportunities for local structural analysis of highly disordered systems. In metallic glasses, the most widely studied amorphous solids by ED methods, these speckled patterns (Figures 11Ci–iv) are proposed to originate from individual atomic clusters, and the assembly of those clusters (Miracle, 2004; Miracle, 2006; Sheng et al., 2006). Although the local atomic arrangements in amorphous metal oxide systems is less established, the structure is proposed to be based on metal-oxide (M-O_x_) polyhedra, where disruptions in long-range order occur by variation in the linking of M-O_x_ polyhedra, or by distortions of the polyhedra themselves that alter bond distances and/or bond angles (Buchholz et al., 2014; Yan et al., 2019).

Pair-distribution function (PDF) analysis (Billinge and Kanatzidis, 2004) is routinely used to extract short-range structural information from highly-disordered or amorphous materials, and is frequently used to study pyrochlore disorder. The PDF yields the probability of finding an atom at an interatomic distance, r, from another atom (Egami and Billinge, 2012), which describes the local atomic environment by providing interatomic bond-lengths and coordination numbers (CN)), averaged over the probed volume. PDF analysis is performed by taking the Fourier transform of the total scattering signal (Bragg + diffuse scatter) to generate a real-space representation, where diffuse and Bragg components of the diffraction data can reveal the local and long-range order (if present) simultaneously (Souza Junior et al., 2021). Although PDF analysis is primarily applied to X-ray and neutron diffraction data, it is also readily applicable to ED data (so-called ePDF analysis). Compared with XRD, ePDF even has an advantage when it comes to lighter atoms because of the stronger electron-matter interactions and the short electron wavelength that enables analysis of atomic-correlations out to high scattering angles (Qmax up to 20 Å−1 is feasible), corresponding to real-space bond lengths of 0.3 Å (Gorelik et al., 2019).

EPDF analysis is most commonly performed on SAED data because it offers a (relatively) large diffracting region (>0.1 μm) that yields statistically averaged information, indicated in the ED data by smooth and continuous halos of intensity (Figure 11Cv) as opposed to the speckled patterns acquired with smaller probes (Figures 11Ci–iv). However, using variable-resolution fluctuation electron microscopy (Voyles and Muller, 2002), a technique for the measurement of MRO, Hwang and Voyles (2011) showed that the diffracted signal of inorganic glasses reaches a statistically averaged representation with just an 11 nm probe. This means that the structure is almost completely homogeneous over that length scale, so ePDF measurements of the averaged amorphous structure can be made from nanometer-scale probes which are compatible with scanning diffraction experiments (4D-STEM). This section discusses scanning ePDF, that can be used to map important structural features across amorphous and highly disordered specimen.

Although X-ray and neutron diffraction-based PDF analysis is widely used in pyrochlore systems, ePDF has not yet been applied to these systems. Authors of a recent review on ePDF analysis shed light on why the technique is underutilized in general, pointing to a lack of access to a user-friendly workflow for handling ePDF processing steps (Souza Junior et al., 2021). Still, ePDF and its close-relative radial-distribution function (eRDF) analysis have been used in many systems, for instance in amorphous SiN (Cockayne, 2007), SiO_2_ (McBride and Cockayne, 2003), SiC (Hirotsu et al., 2001; Ishimaru et al., 2002; Ishimaru, 2006; Ishimaru et al., 2008)), and more software tools for ePDF analysis are being developed all the time (e.g., eRDF Analyzer (Shanmugam et al., 2017), RDFTools (Mitchell et al., 2012), PASAD (Gammer et al., 2010), SUePDF (Tran et al., 2017), ePDFsuite (Nanomegas, Belgium). Thorough overviews of ePDF analysis are given in references (Gorelik et al., 2019; Souza Junior et al., 2021).

When the electron probe is brought down to the angstrom scale (Figure 11Ci, information about the local atomic arrangement of clusters can be extracted. ABED has been broadly applied to metallic glasses (Hirata et al., 2011; Hirata et al., 2013; Zhu et al., 2017), and to oxides (Hirata et al., 2016), however application to amorphized mixed-metal oxides is largely absent from the literature. This is likely because metal-oxides are not ideal glass formers (Lim et al., 2015); however, their structure is still technologically important, (Yan et al., 2019), especially for understanding radiation-induced disordering processes. Recently, Raman (Tracy et al., 2016) and STEM-EELS (Sachan et al., 2017b) spectroscopies have indicated that the local atomic arrangement within amorphous pyrochlore is not fully random as previously thought, but may instead exhibit local ordering similar to that of the disordered phase (Shamblin et al., 2018). Hirata and coworkers, who have made substantial advancements in determining the local structure of amorphous materials through ABED, suggests that a combination of global (e.g., statistically averaged PDF analysis) and local (e.g., ABED) analysis is necessary to provide the overall picture of amorphous structures. PDF analysis cannot adequately reveal individual local structures, although the overall statistical information can be obtained accurately. Therefore, the higher spatial resolution of ABED compared to that of PDF is necessary to directly obtain local information (Hirata, 2021). Additionally, ABED combined with the scanning function of STEM allows the spatial extent and distribution of local structures to be understood.

#### Precession Electron Diffraction

In most (S)TEM techniques, data interpretation is complicated by multiple scattering which commonly occurs due to the strong interaction of electrons with the crystal potential, even in thin specimens (<100 nm). This ‘dynamical’ diffraction perturbs the relative intensities of Bragg reflections and diffuse scatter in ED, and leads to inaccurate measurement of CN in ePDF analysis (Ishimaru, 2006). The first line of defense to minimize multiple scattering is optimization of specimen thickness and/or electron energy (Gorelik et al., 2019), however, many other methods have been devised (Eggeman et al., 2012) specifically to avoid its effect on the relative intensities of Bragg peaks in ED patterns. PED (Ishimaru et al., 2002) is one of these methods, whereby the incident electron beam (often set to ΝΒED conditions), is tilted and precessed to form a conical electron probe at the specimen. PED can be used to identify crystal structure, determine the local crystal orientation (Bowman et al., 2020), or to investigate crystal texture, grain rotation, and strain. Compared with a steady (unprecessed) beam, precession provides many more reflections by sampling additional layers of reciprocal-space (both the zero-order and higher-order Laue Zones, ZOLZ and HOLZ, respectively) allowing for greater sensitivity to local orientation changes as well as changes in lattice parameter. It also integrates the intensities through the Bragg condition over the precession cycle, averaging out relative intensity fluctuations due to dynamical scattering (Moeck and Rouvimov, 2010; Midgley and Eggeman, 2015).

#### Convergent-Beam Electron Diffraction

Until this point, the diffraction techniques discussed have utilized parallel (*α* ≈ 0), or quasi-parallel (small *α*) illumination that produce localized diffracted signals originating from a broad specimen area. CBED requires convergent illumination, where the electron beam is focused into a probe that impinges the specimen over a range of angles (Williams and Carter, 2009b) and delocalizes the diffracted signal into discs that may contain complex, and information-rich intensity distributions (Zuo et al., 2017b). Generally, the convergence angle used for CBED is several times larger than what is used in μ/N/ΑBED, but still significantly smaller than that used in an aberration-corrected STEM. The convergent illumination condition generates a spherical electron-wavefront capable of sampling a greater portion of reciprocal-space, thus, CBED is better suited than parallel-beam ED approaches for involved crystallographic studies because it enables unique determination of the point group from one or few zone axes, and with analysis of the systematic absences it is possible to uniquely determine most space groups. The complex intensity distributions within the Bragg discs, and 3D information in the higher-order Laue zone (HOLZ) lines (Zou et al., 2011) provide additional signatures of the underlying crystal symmetry. Positions of the HOLZ lines are sensitive to the unit cell parameters, so deviation from a standard can be used to probe any local unit cell distortions. See (Zuo et al., 2019) for an in-depth discussion of S-BED techniques and their utility.

## Linking Real and Reciprocal-Space Information With 4D-STEM

4D-STEM refers to a broad range of scanning-based diffraction techniques that generate a four-dimensional diffraction dataset, where each probe position in real-space is associated with a diffraction pattern in reciprocal-space (Zuo et al., 2019). In addition to providing a direct link between these two spaces, 4D-STEM is a powerful alternative to STEM imaging (Figure 11A) because it utilizes a “universal (pixelated) detector” (Figure 11B) such that the full angular range of electron scatter is collected simultaneously. In this 4D dataset, information can be parsed as a function of the scattering vector (k_x_,k_y_) in reciprocal space and probe position (x,y) on the sample (Hachtel et al., 2018) (Figure 11C).

### Terminology, Acquisition and Challenges

The presence of the term ‘4D-STEM’ in the literature is fairly new, largely due to a lack of agreed upon terminology. In review of 4D-STEM techniques and applications, C. Ophus (Ophus, 2019) provides early examples of work appearing under different names despite their similar experimental setups (e.g., position-resolved diffraction, spatially-resolved diffractometry, momentum-resolved STEM, scanning electron nanodiffraction, pixelated STEM, nanobeam scanning diffraction, nanodiffraction mapping, STEM diffraction mapping, etc.). Nonetheless, variations of 4D-STEM experiments have been widely used under various aliases over the past ∼20 years with increasing ease and popularity as detector technology advances.

4D-STEM is performed under small-beam diffraction conditions (described in an earlier section) since size the of the beam on the sample provides an upper limit to the achievable spatial resolution of the structural information maps in most cases. The convergence angle, determined by the probe-forming aperture and settings of the electron optics, controls the probe size on the specimen and the size of the BF and Bragg discs in the diffraction plane, where an inherent tradeoff exists between the two. Some important factors to consider in these experiments are:

1) the extent of overlap between Bragg discs which will determine what types of analyses can be performed (e.g., well-separated discs are required for phase, orientation, and strain mapping, and disc interference is needed for ptychographic reconstruction of atomic potentials).
2) the spatial sampling of the probe which in the case of undersampling, determines the spatial resolution and the field-of-view (FOV) of structure maps, and can exclude certain types of analyses (e.g., position-averaged CBED, (PACBED)) for which oversampling is required.
3) the dwell times and probe current, which will depend on how well the sample tolerates the electron beam. See (Egerton, 2019) for a review of electron beam damage and (Bustillo et al., 2021) specific to 4D-STEM experiments.
4) The number of pixels over the scanned area, and on the detector. For example, a spatial scan with 1 k × 1 k points recording data from a 512 × 512 pixel detector would occupy ∼977 GB when stored in 32-bit integer format (Nord et al., 2020).

See (Nord et al., 2020; Paterson et al., 2020) for guides on 4D-STEM data acquisition and processing.

### Applications of 4D-STEM

**FIGURE 12:**
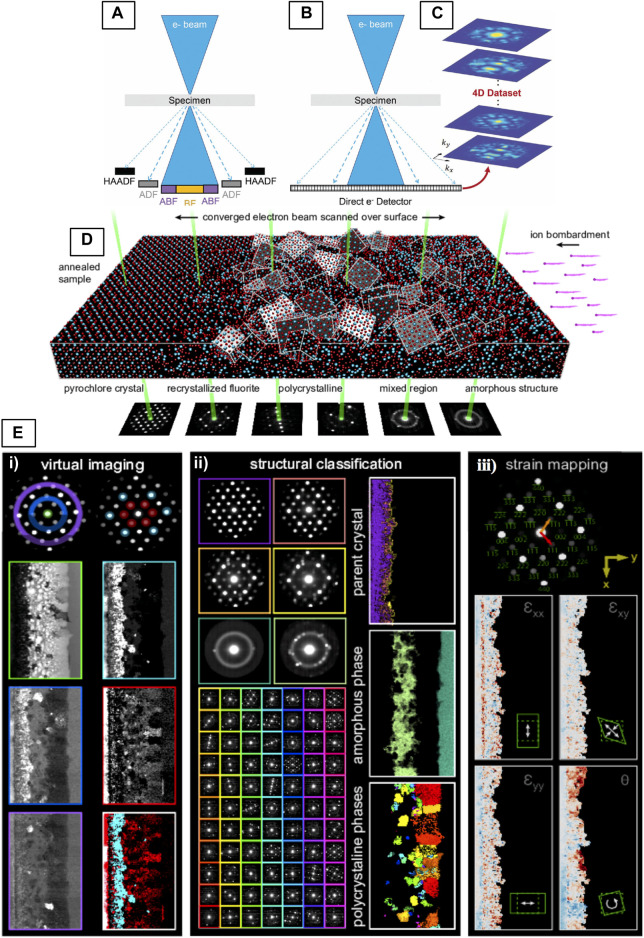
**(A–C)** Illustration of conventional and 4D-STEM and **(D,E)** 4D-STEM experimental geometry, and multimodal data analysis with py4DSTEM. **(A)** In conventional STEM, signal is integrated over different angular regions of the diffraction plane. **(B)** In 4D STEM, an entire 2D CBED pattern is recorded at each probe position of a 2D-STEM raster, resulting in a **(C)** 4D dataset, Reproduced from (Levin et al., 2020b). **(D)** The experimental setup showing a pyrochlore-structured Gd_2_Ti_2_O_7_ (GTO) sample that was irradiated and subsequently annealed showing gradient in structure from fully-ordered to amorphous (Savitzky et al., 2021). **(E)** Examples of various types of measurements that can be made in post-processing from the 4D-STEM dataset acquired in **(D)**. Reproduced from reference (Savitzky et al., 2021).

The ability to collect the full scattering range, and associate diffraction data with positions across the specimen leads to a broad range of applications. Barring some specific acquisition requirements that may complicate or exclude data analysis for certain applications, the 4D dataset contains all information needed to perform multiple types of analysis. A single 4D-STEM experiment enables a range of measurements that can be performed in post-processing. Rather than providing a comprehensive overview of possible techniques, which already exists (Ophus, 2019), we will introduce select 4D-STEM techniques that are particularly useful for the analysis of disordered crystalline systems, specifically for the types of disorders described in previous sections. These include virtual imaging, structure classification followed by crystalline/semi-crystalline orientation, phase and strain mapping, as well as analyses specific to amorphous materials such as SRO/MRO analysis with STEM-PDF/RDF, and STEM-FEM. 4D-STEM has not been widely applied to pyrochlores and fluorites, fortunately there is one detailed example of its application to a pyrochlore-structured Gd_2_Ti_2_O_7_ (GTO) in the literature, used to illustrate the multimodal analysis of 4D datasets using the py4DSTEM software suite (Savitzky et al., 2021). In this example, a GTO single-crystal was amorphized through an irradiation treatment, then partially recrystallized through annealing. The sample contains a band of semi-crystalline GTO sandwiched between single-crystal and amorphous states. Figure 12B shows the complex, multi-phase structure of this sample, and also demonstrates the experimental setup of the 4D-STEM experiment where an SBED Small-Beam Electron Diffraction - Microbeam, Nanobeam, Angstrom Beam and Convergent Beam Electron Diffraction pattern is acquired for each probe position on the GTO specimen (Figure 12B). Figure 12C shows a few of types of analyses that have been performed on this GTO dataset.

#### Virtual Imaging

**FIGURE 13:**
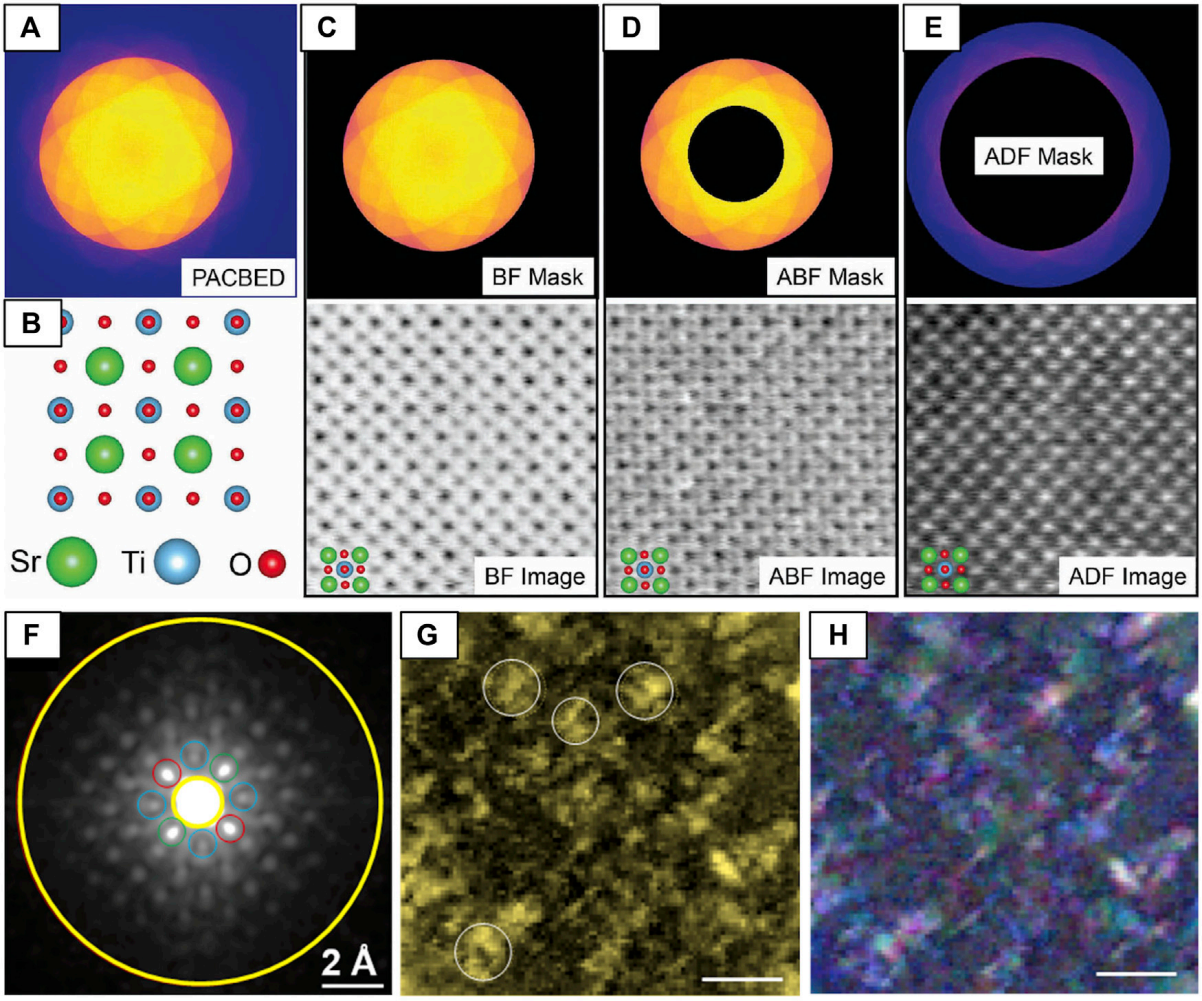
Using 4D-STEM datasets to reconstruct virtual images **(A–E)** of SrTiO3 at atomic-resolution and **(F–H)** of severe lattice distortions in an HEA using the difference Cepstrum (dCp) transform to select only diffuse electron scatter contributions. **(A)** A 4D-STEM PACBED dataset acquired from SrTiO_3_ [001] and the **(B)** crystal structure of SrTiO_3_ [001]. Masks (top row) can be applied to the 4D dataset to produce **(C)** BF **(D)** ABF and **(E)** ADF images, reproduced from reference (Levin et al., 2020b). The **(F)** (dC_p_) averaged across the scanned region. A **(G)** virtual-image formed from the total diffuse scattering signal integrated over yellow annulus in **(F)** and an **(H)** composite virtual-image of the blue, green, and red signals in the dC_p_ showing the complex structural distortions of the HEA. Scale bars in G and H are 40 nm, reproduced from (Shao et al. 2021).

The most straightforward analysis that can be performed on a 4D-STEM dataset is to integrate the diffracted signal within a (usually circular or annular) mask generated in reciprocal-space to construct a 2D-STEM image (Levin et al., 2020b). The ability to flexibly select signals in reciprocal-space makes it possible to reconstruct images equivalent to any of the traditional STEM detectors (see STEM Imaging Modes), even with atomic resolution (Figures 13C–E) (Hachtel et al., 2018; Levin et al., 2020b). The left column of images in Figure 12ei (Savitzky et al., 2021) shows an example of virtual imaging with circular and annular masks at low magnification, used to generate virtual-BF (green) and virtual-DF (blue/purple) images of the irradiated GTO sample, ED patterns in the <110> projection. The second column of Figure 12Ei shows the unique power of virtual imaging, where specific diffraction spots, sets of spots, or other diffraction fine-structure can be selected to form images that may be otherwise impossible to generate through DF imaging techniques. Here, the virtual-DF images are constructed from sets of cation ordering reflections (Lian et al., 2003) that correspond to the pyrochlore structure (red), and from a set of reflections shared by both pyrochlore and fluorite (blue), demonstrating how local regions of cation ordering can be imaged by filtering electrons from the diffraction pattern based on their scattering vectors. Another approach to mapping cation-ordering achieved by template matching is described in the following Structure Classification, Orientation, and Strain Mapping. In theory, imaging of the anion-ordered regions could be achieved in a similar manner by selecting spots with indices *h* + *k + l =* 4*n* (i.e., 022 and 422) (Lian et al., 2003) to form virtual-DF images. However, the scattering from oxygen is likely much weaker and may require methods to improve the signal-to-noise ratio.

By the same logic, virtual imaging could also be used to image locally-ordered domains (O’Quinn et al., 2020) in heterogeneously disordered pyrochlores (e.g., to reveal the formation of Weberite-type (O’Quinn et al., 2020; Shamblin et al., 2016), or bixbyite (C-type) structural units (Reid et al., 2012). It could also be used to investigate the spatial the origins of diffuse electron scatter (see Diffraction Fine-Structure) from the specimen, however, this presents several challenges. A more sophisticated approach is achieved by Cepstral STEM (Shao et al., 2021), which is a new 4D-STEM technique for imaging severe lattice distortions within disordered crystals using fluctuations in electron diffuse scatter that arise due to crystal disorder. Cepstral analysis is a sensitive signal processing technique for detecting weak harmonic signals by performing a Cepstral transform of the coherent NBED pattern. The Cepstrum transform has been applied previously to ED patterns for lattice strain analysis based on Bragg scatter (Padgett et al., 2020). Recently, the difference Cepstrum (dC_p_) was developed, which represents the difference between the Cepstral transforms of local NBED patterns and the region-averaged NBED pattern within a 4D dataset (Shao et al., 2021). The dC_p_ separates electron diffuse scattering from Bragg diffraction so the diffuse signal can be analyzed to determine the distortive part of electron scattering potential. This part can then be imaged via construction of a virtual-DF image (Figure 13) as in the previous example, only by masking the dC_p_ rather than the NBED pattern directly. The sharp harmonic signals detected by Cepstral STEM make numerical quantification of lattice distortions and their mapping possible. Figure 13 shows the dC_p_ of a high-entropy alloy, where the composite virtual-image from select signals in the dC_p_ demonstrate the complex structural distortions of and HEA. See (Ophus, 2019) for more applications of virtual imaging to various systems.

#### Structure Classification, Orientation, and Strain Mapping

For structure classification, phase, orientation, and strain mapping applications, precise determination of Bragg disc positions is critical, thus, the discs must be well-separated and well-defined so automated analysis can be carried out to determine their locations. The overlap of discs can be prevented by reducing the convergence angle such that the distance between the Bragg spots is at least twice the Bragg angle (θ_B_ ≈ few mrad) which can be achieved by ensuring α < θ_B_. Phase and orientation mapping rely on a template matching approach, where stacks of simulated diffraction data are matched to the experimental data to determine the local phase, orientation, or strain at each probe position (Rauch et al., 2010). Figure 12Eii shows classification of the GTO sample into three distinct structural phases, the pyrochlore (parent) structure, the recrystallized and amorphous regions, which can then be mapped to observe their spatial distributions. 4D-STEM has been used to quantify and map the degree of cation disorder in pyrochlore materials at the nanoscale by quantifying the intensity ratio of pyrochlore superlattice reflections to the main fluorite reflections for each pattern in the scanning NBED (4D-STEM) dataset can be generated (Janish et al., 2019). Again, a template matching approach is implemented to match each experimental pattern with a dictionary of simulated diffraction patterns (Allen et al., 2015) generated for calculated structures with varying degrees of disorder (Janish et al., 2019; Janish et al., 2020).

#### Analysis of Amorphous Materials

**FIGURE 14:**
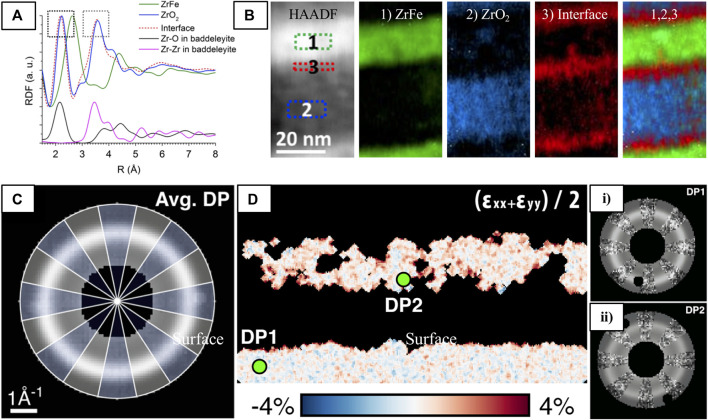
Analysis of amorphous materials with 4D-STEM to map **(A,B)** the atomic arrangement of ZrO_2_/ZrFe multilayers and **(C,D)** strain in the irradiated GTO sample. **(A)** RDFs taken from the amorphous ZrFe (green), ZrO_2_ (blue), and interfacial layers (red). The interface RDF is similar to the ZrO_2_ RDF with shifts in the first (0.04 Å, left) and second (0.06 Å, right) peaks indicated by black boxes. Comparison with the calculated Z-O (black) and Zr-Zr (pink) partial RDFs based on monoclinic crystalline baddeleyite ZrO_2_ demonstrate that the SRO of ZrO_2_ and the interfacial layer is similar to baddeleyite, and the shifts to reduced atomic distances can be explained by Fe replacing Zr in the ZrO_2_ clusters at the interface. **(B)** From left to right, STEM-HAADF image of the ZrO_2_/ZrFe layered structure, and maps corresponding to regions 1) ZrFe, 2) ZrO_2_, 3) their interface and their composite image. **(C)** Elliptical fits (shown under grey wedges) of the averaged ED pattern (blue wedges) of the amorphous GTO from Figure 12. **(D)** A strain map of the amorphized regions and (i,ii) ED patterns from two distinct regions indicated in **(D)** where the fitted and raw data alternate azimuthally as in **(C)**. Reproduced from **(A,B)** (Mu et al., 2016b) and **(C,D)** (Savitzky et al., 2021).

In a previous section, we saw the utility of the ePDF/eRDF for analyzing the structure of amorphous materials from diffraction patterns. This approach becomes much more powerful when coupled with a scanning beam in order to map out real-space distributions of SRO (Lu and Gauntt, 2013). Most implementations of so-called “STEM-PDF” (Mu et al., 2021) or “STEM-RDF” (Mu et al., 2016a) require some type of multivariate statistical analysis (MVSA) used to mine large 4D datasets to identify relevant co-varying variables in order to map statistically significant structural components (Lu and Gauntt, 2013; Mu et al., 2019). For example, to disentangle the local atomic bonding and packing information mixed in NBED data to characterize nanoscale heterogeneous amorphous materials such as nanoglass (Mu et al., 2021). Or to reveal interfacial layers from an amorphous ZrO_2_/Zr_0.2_Fe_0.8_ mutilayer system with unique atomic arrangements that could not be seen with EDX or EELS (Figures 14A,B) (Mu et al., 2016a). As in other 4D-STEM approaches, the size the of the beam on the sample will limit the spatial resolution of structural information maps. A small beam (down to 2 nm), can be used, but speckled regions of the pattern that appear as the beam size is reduced (recall Figure 11C) must be removed with masking in analysis (McBride and Cockayne, 2003). Overall, the nm-scale spatial resolution of STEM-PDF is a dramatic improvement to the 10 μm spatial resolution of x-ray synchrotron PDF mapping (Billinge, 2019).

Like ePDF, fluctuation electron microscopy (FEM) is used to characterize the structure of amorphous materials in the (S)TEM. However, in contrast to ePDF which is ideal for measuring pair-correlations out to distances of the first few shells of neighboring atoms, it is better suited for measuring MRO which extends beyond the first few shells. It has been shown that the two-body correlations are not sufficient to distinguish between competing structural models of some amorphous materials (Treacy and Borisenko, 2012), therefore access to MRO is necessary for full structural analysis. It can be performed in both TEM and STEM mode, although STEM mode facilitates easier convergence-angle adjustment to investigate different sizes of atomic clusters (Ophus, 2019; Savitzky et al., 2021) as well as to determine correlation lengths of locally-ordered regions (Voyles and Muller, 2002; Voyles et al., 2011). When these clusters deviate from a fully random distribution, they lead to “speckles” in the amorphous halo as described previously, FEM provides structural information by quantification of the degree of variability as a function of scattering angle and probe size (Treacy et al., 2005; Hilke et al., 2019). Like ePDF and ABED analyses, FEM has primarily been applied to metallic glasses however, there are some examples of its use in oxide systems. For example, to study crystal nucleation in TiO_2_-SiO_2_ glasses upon heat treatment (Rezikyan and Moore, 2020), and to analyze structural disordering in ion-irradiated amorphous zircons (Zhao et al., 2010). For a review of FEM theory and development see (Voyles et al., 2011).

Figure 12Eiii demonstrates crystalline strain mapping from determination of Bragg disc position. Like crystalline materials, a change of the average atomic spacing in an amorphous material will result in a change in the radius of the diffuse halo. When this halo is fit to an elliptical function local strain can be measured (Ebner et al., 2016) and mapped (Savitzky et al., 2021; Gammer et al., 2018) if acquired with a scanning beam. The py4dSTEM implementation of this analysis (Savitzky et al., 2021) on the amorphized region of the GTO sample is shown in Figures 14C,D, where the $1/2\left(\varepsilon_{xx}+\varepsilon_{yy}\right)$ map shows the local dilation, which is amplified in the vicinity of amorphous-polycrystal interfacial regions. This method has also been combined with *in situ* tensile testing to reveal the accumulation of strain under mechanical deformation in a bulk metallic glass (Gammer et al., 2018).

#### Other Applications

Many variations of 4D-STEM exist, for example, to map electromagnetic fields (Hachtel et al., 2018). Other applications aim to increase the sensitivity to light elements (Hachtel et al., 2018; Ahmed et al., 2020) and to retrieve lost electron-wave phase information to achieve super-resolutions (Jiang et al., 2018; Chen et al., 2021). A detailed account of the various 4D-STEM methods can be found in (Ophus, 2019).

#### 4D-STEM Resources

4D-STEM approaches are becoming increasingly accessible with the development of more suitable hardware, larger data storage capacity, more powerful computation, and the continued development of software packages for simulation and analysis that facilitates easier analysis workflows. The analysis of such large, multidimensional datasets requires powerful computers and specialized software, many of which are home-grown in various labs around the world. Some available packages are Hyperspy, py4DSTEM, LiberTEM, Pycroscopy, pixSTEM, and the Cornell Spectrum Imager plugin for ImageJ. 4D-STEM simulation software (Pelz et al., 2021) is also critical component of analysis of 4D datasets.

## Summary and Outlook

In this review, we hoped to demonstrate the utility of (S)TEM for characterization of pyrochlore, fluorite, and other disorder-harboring complex oxide systems. Particularly to address the challenge of characterizing the various types of local transformations they adopt that can easily go undetected without the ability to assess the structure on a highly localized scale. In summary, the capabilities of (S)TEM to serve this end are as follows:

1) The positions of atomic columns can be extracted with picometer precision in both CTEM and STEM imaging with the assistance of image analysis software, which enables the analysis of small atomic displacements and provides the basis for at least one of the many approaches to strain mapping presented here.
2) The flexible illumination optics of (S)TEM systems offer unparalleled control over the volume (and position) of the electron probe on the specimen. This is especially useful for diffraction analysis, where the probe volume can be tuned across the sub-nanometer to tens of nanometers range to approach the length-scale of ordering/disordering in these systems (e.g., to probe short-, medium- or long-range order), revealing new signatures of local-ordering not accessible with larger probes.
3) The sensitivity of (S)TEM signals to different elemental atomic numbers can also be tuned, either through control of pre-specimen aberration-correction optics or by controlling the angular range of collected scatter post-specimen, which is key for probing the oxygen sublattice in the presence of heavy metals.
4) Small-area diffraction techniques combined with a scanning beam (4D-STEM) expand the utility of diffraction experiments by linking localized diffraction information with a real-space position on the specimen, enabling structural features of interest to be mapped without the need for painstaking point-by-point manual acquisition.

Going forward, as 4D-STEM is increasingly used in tandem with *in situ* and *in operando* studies, the dimensionality of these already rich, multidimensional datasets will extend into the temporal domain, as well as along the axis of the applied stimuli. Additionally, these structure-function studies could be supplemented with chemical information from EDXS and EELS signals, in the latter case, yielding the BF disc to the EELS spectrometer through a physically modified, “hollow” detector (Song et al., 2018). As more data can be acquired at once to achieve *n*-dimensional datasets, software tools will advance in step to both increase user-friendliness and accessibility, as well as to provide more advanced and sophisticated analyses that can fully utilize and correlate each dimension of data. These capabilities will reveal the true value of the modern (S)TEM in providing fundamental insights into the dynamic and functional behaviors of complex systems.
